# Substituent Effects and Mechanistic Insights on the Catalytic Activities of (Tetraarylcyclopentadienone)iron Carbonyl Compounds in Transfer Hydrogenations and Dehydrogenations

**DOI:** 10.1021/acs.organomet.3c00284

**Published:** 2023-10-05

**Authors:** Bryn K. Werley, Xintong Hou, Evan P. Bertonazzi, Anthony Chianese, Timothy W. Funk

**Affiliations:** † Department of Chemistry, Gettysburg College, Gettysburg, Pennsylvania 17325, United States; ‡ Department of Chemistry, Colgate University, Hamilton, New York 13346, United States

## Abstract

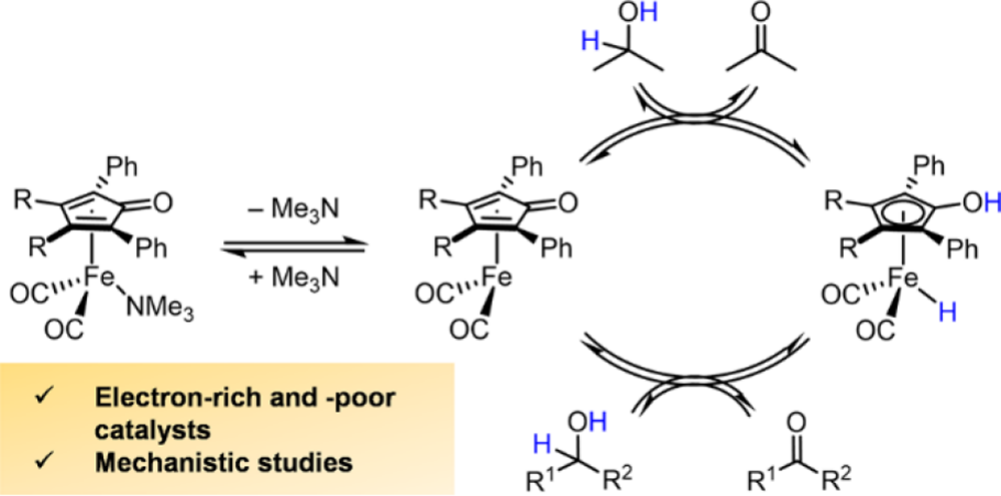

(Cyclopentadienone)iron carbonyl compounds are catalytically active in carbonyl/imine reductions, alcohol oxidations, and borrowing hydrogen reactions, but the effect of cyclopentadienone electronics on their activity is not well established. A series of (tetraarylcyclopentadienone)iron tricarbonyl compounds with varied electron densities on the cyclopentadienone were prepared, and their activities in transfer hydrogenations and dehydrogenations were explored. Additionally, mechanistic studies, including kinetic isotope effect experiments and modifications to substrate electronics, were undertaken to gain insights into catalyst resting states and turnover-limiting steps of these reactions. As the cyclopentadienone electron density increased, both the transfer hydrogenation and dehydrogenation rates increased. A catalytically relevant, trimethylamine-ligated iron compound was isolated and characterized and was observed in solution under both transfer hydrogenation and dehydrogenation conditions. Importantly, it was catalytically active in both reactions. Kinetic isotope effect data and initial rates in transfer hydrogenation reactions with 4′-substituted acetophenones provided evidence that hydrogen transfer from the catalyst to the carbonyl substrate occurred during the turnover-limiting step, and NMR spectroscopy supports the trimethylamine adduct as an off-cycle resting state and the (hydroxycyclopentadienyl)iron hydride as an on-cycle resting state. In transfer dehydrogenations of alcohols, the use of electronically modified benzylic alcohols provided evidence that the turnover-limiting step involves the transfer of hydrogen from the alcohol substrate to the catalyst. The trimethylamine-ligated compound was proposed as the primary catalyst resting state in dehydrogenations.

## Introduction

**Figure 1:**
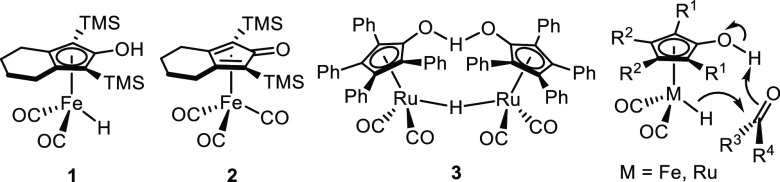
Knölker’s iron hydride (**1**); its air-stable, tricarbonyl derivative (**2**); Shvo’s catalyst (**3**); and the concerted outer-sphere mechanism through which they transfer hydrogen.

As chemists strive to develop catalytic processes using sustainable metals, interest in exploring the reactivity of (cyclopentadienone)iron carbonyl compounds has grown. They were first prepared in the 1950s,^1−5^ but their catalytic potential was not realized until the 21st century, when Casey and Guan showed that Knölker’s iron hydride (**1**)^6^ reduced ketones and aldehydes under a hydrogen atmosphere.^7^ Experimental^8,9^ and computational^10,11^ studies of **1** support it reacting with carbonyls through an outer-sphere, concerted, bifunctional mechanism, where both the Fe center and the cyclopentadienone carbonyl are involved in hydrogen transfer (Figure 1). There is evidence that the diruthenium bridging hydride known as Shvo’s catalyst (**3**) reacts through a similar mechanism.^12−14^

During the past decade, a variety of oxidative and reductive catalytic processes using (cyclopentadienone)iron carbonyl compounds have been developed, including reductions of carbonyls and imines, oxidations of alcohols, and borrowing hydrogen reactions.^15−17^ Additionally, it has become clear that cyclopentadienone substitution affects the catalyst activity. For example, changing the substitution on the ring fused to the cyclopentadienone affects its activity in alcohol oxidations,^18−21^ aldehyde and ketone reductions,^9,22−29^ imine reductions/reductive aminations,^30−33^ and borrowing hydrogen processes.^34−39^ Unfortunately, there have been few studies systematically exploring the underlying effects of these modifications in large part because structural modifications to the cyclopentadienone often affect both its steric and electronic properties, making it difficult to develop structure–activity relationships.

We illustrated that (3,4-diphenylcyclopentadienone)iron carbonyl compounds were active in transfer hydrogenations and dehydrogenations,^40^ and we concluded that these compounds would serve as a useful template to study how cyclopentadienone electronics affect catalyst activity. The electron density of the cyclopentadienone could be tuned by modifying the para positions of the two appended aromatic rings with minimal changes to its overall size, especially in the region close to the Fe center. Ultimately, our goal was to probe how cyclopentadienone electronics affect transfer hydrogenations and dehydrogenations and gain mechanistic insights into the catalytic cycle of these processes.

**Scheme 1:**
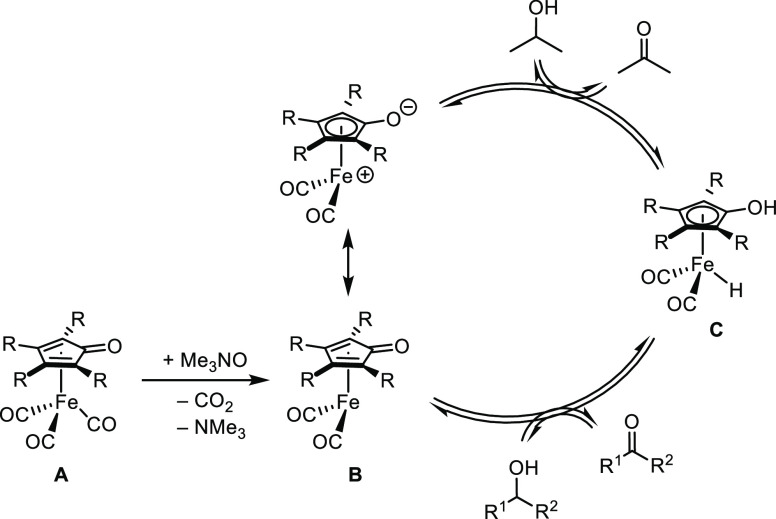
Typical Proposed Catalytic Cycle for Transfer Hydrogenation and Dehydrogenation with (Cyclopentadienone)iron Carbonyl Compounds Activated with Trimethylamine *N*-oxide

The typical proposed catalytic cycle is shown in Scheme 1, where moving clockwise corresponds to transfer hydrogenation and counterclockwise corresponds to transfer dehydrogenation. Activation of tricarbonyl compound **A** with trimethylamine *N*-oxide in solution forms unsaturated species **B**, which is catalytically active. Iron hydride **C**, which is also catalytically active, is generated when **B** reacts with an alcohol.

## Results and Discussion

### Catalyst Preparation

**Scheme 2:**
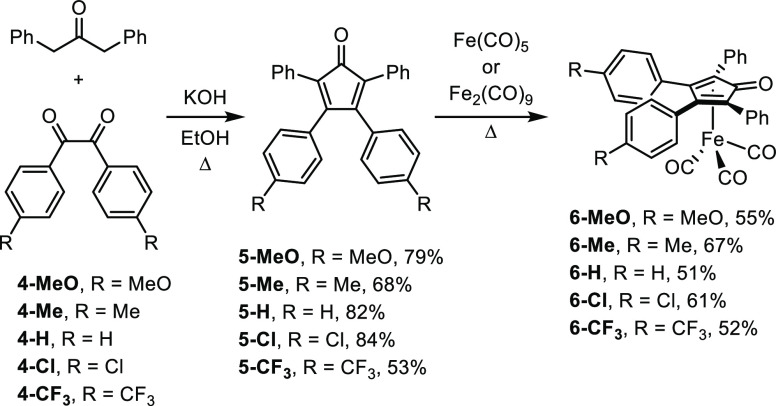
General Synthetic Approach to (Cyclopentadienone)iron Carbonyl Compounds

The general synthetic approach to our five targeted iron compounds is shown in Scheme 2. Using known procedures, benzil or a substituted benzil (**4**) was coupled with 1,3-diphenylacetone to form the cyclopentadienones (**5**),^41^ which were reacted with an iron carbonyl compound to generate (cyclopentadienone)iron compounds **6**.^5,42−44^ Two substituted benzils—**4-Cl** and **4-CF**_**3**_—were prepared in two steps from inexpensive precursors.^45^ All of the (cyclopentadienone)iron tricarbonyl compounds **6** were air-stable solids that were stored in the dark under air at 4 °C, and no decomposition was detected after one year under these storage conditions.

### Catalytic Activity Comparison

**Figure 2:**
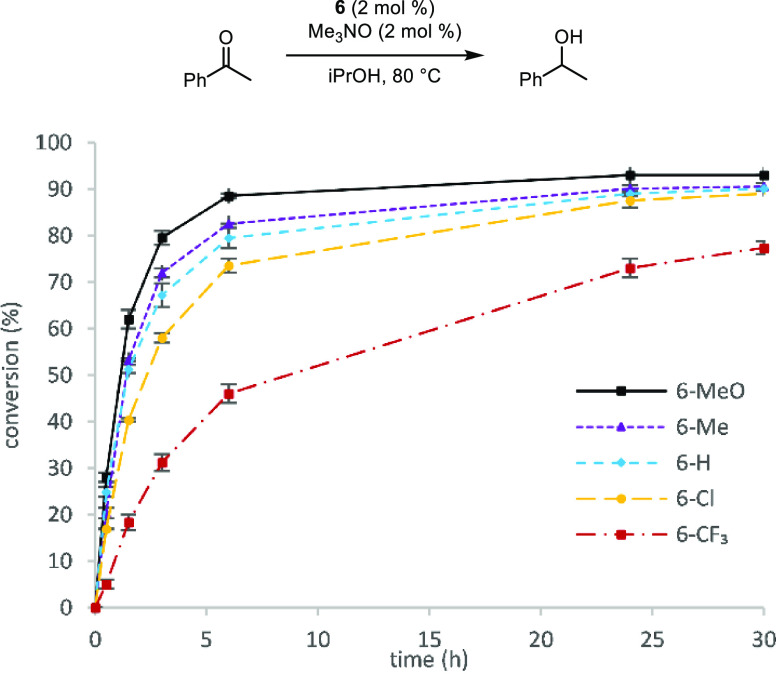
Conversion (%) vs time (h) for the reduction of acetophenone using catalysts **6-MeO**, **6-Me**, **6-H**, **6-Cl**, and **6-CF**_**3**_. Plotted points are averages of at least two runs. Conversions were determined by GC relative to biphenyl.

The catalytic activity of all five (cyclopentadienone)iron tricarbonyl compounds (**6**) was explored in both transfer hydrogenation and dehydrogenation reactions, and the results from the transfer hydrogenation of acetophenone in isopropanol are shown in Figure 2. The conversions over time were consistent between runs as is evident from the small error bars, and the data illustrated a correlation between catalyst electronics and conversion—higher conversions occurred with more electron-rich substituents. At longer reaction times, conversions using **6-Me**, **6-H**, and **6-Cl** converged and were slightly below the conversion with **6-MeO**. Although it was the least active, **6-CF**_**3**_ still turned over 24 h after the reaction began.

**Figure 3:**
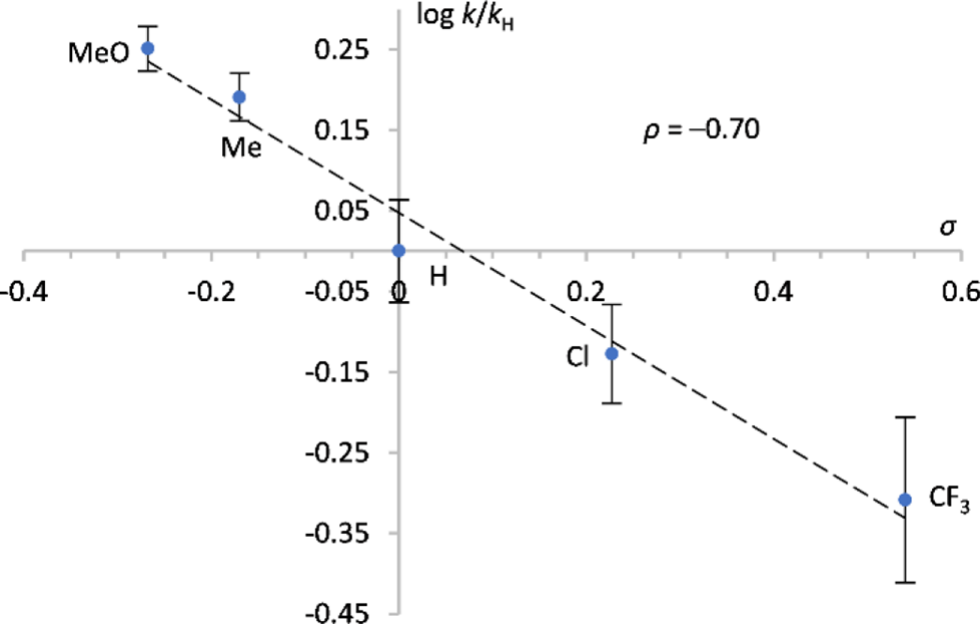
Hammett plot (log[*k*/*k*_H_] vs σ) for the transfer hydrogenation of acetophenone using catalysts **6-MeO**, **6-Me**, **6-H**, **6-Cl**, and **6-CF**_**3**_.

Initial rate data was collected for the reduction of acetophenone with the five catalysts **6**, and a Hammett plot showed a linear free energy relationship between the logarithm of the initial reaction rates (relative to **6-H**) and Hammett’s substituent constants (Figure 3).^46^ The plot supports the trend observed in Figure 2: the more electron-rich the catalyst, the faster the initial rate. The reaction constant of −0.70 is consistent with the development of a positive charge or a decrease in electron density occurring on the catalyst during the slow step in the catalytic cycle.

**Figure 4:**
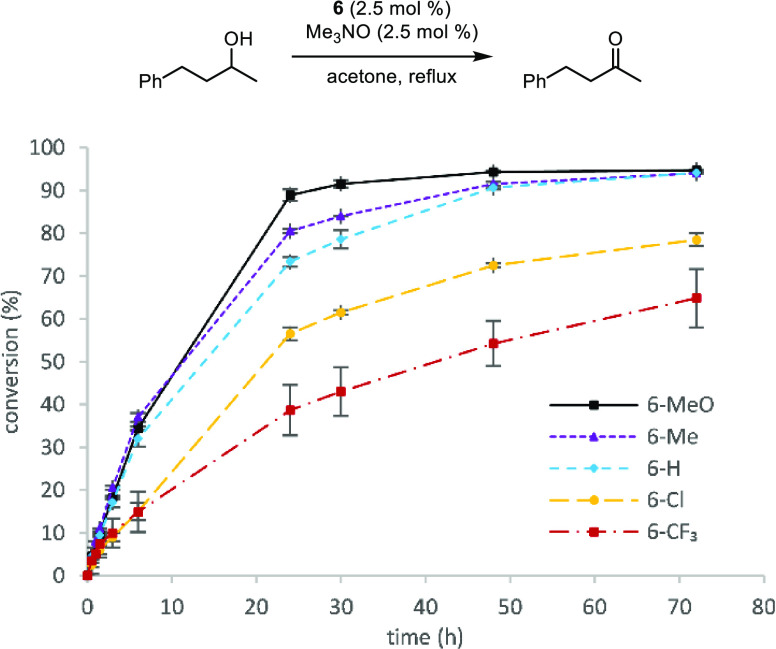
Conversion (%) vs time (h) for the transfer dehydrogenation of 4-phenyl-2-butanol using catalysts **6-MeO**, **6-Me**, **6-H**, **6-Cl**, and **6-CF**_**3**_. Plotted points are averages of at least two runs. Conversions were determined by GC relative to biphenyl.

The same data was collected for the transfer dehydrogenation of 4-phenyl-2-butanol in acetone using the same five catalysts (Figure 4). Overall, the oxidation reactions occurred more slowly than the carbonyl reductions, but the same general trend was observed. After 24 h, higher conversions correlated to higher cyclopentadienone electron density and all catalysts remained active. Catalysts **6-H** and **6-Me** afforded the same conversion obtained with **6-MeO** after 3 days. Attempts to confidently establish a linear free energy relationship using initial rates were unsuccessful due to variation in the initial rates. The initial rates for **6-MeO**, **6-Me**, and **6-H** overlapped during the first 10–20% conversion, and the data overlapped for **6-Cl** and **6-CF**_**3**_ at low conversions too. Additionally, the error was relatively large at these early conversions, which may be due to the low solubility of trimethylamine *N*-oxide in acetone. Interestingly, the conversions were consistent at 24 h and beyond.

Overall, (cyclopentadienone)iron tricarbonyl compounds with electron-rich substituents were more active than those bearing electron-withdrawing groups in both transfer hydrogenation and dehydrogenation. The same trend was observed when analogous derivatives of Shvo’s catalyst (**3**) were used in the formic acid-catalyzed disproportionation of aldehydes,^47^ the transfer hydrogenation of aldehydes and ketones using formic acid,^48^ and the racemization of secondary alcohols.^49^ In the disproportionation of aldehydes to esters, it was proposed that electron-donating groups accelerated reaction rates by increasing the basicity of the cyclopentadienone carbonyl oxygen atom, which promoted a stronger hydrogen bond with the incoming alcohol and decreased the energy of hydrogen transfer to the catalyst from a hemiacetal.^47^

Additionally, (cyclopentadienone)iron carbonyl complexes with electron-rich cyclopentadienones bearing amines were shown to be more active than similar catalysts in ketone reductions and reductive aminations using isopropanol and H_2,_^31,50,51^ which is consistent with our results. DFT studies showed low barriers for hydrogen transfer from the catalyst to carbonyl/imine, and the activation of iron-bound dihydrogen in reductions with H_2_ was proposed to be the turnover-limiting step.^31,51^ Dihydrogen is not used in our transfer hydrogenations, making a comparison difficult. Additionally, the introduction of the aliphatic amines or diamine ring led to cyclopentadienone steric changes, so making a firm conclusion based exclusively on electronic factors was challenging.

A computational study by Li and co-workers proposed that decreasing electron density on the cyclopentadienone should increase the rate of benzaldehyde reduction,^52^ which is inconsistent with our data. They identified hydrogen transfer from the (hydroxycyclopentadienyl)iron hydride (e.g., **1**) to the aldehyde to be the turnover-limiting step, and their calculations were focused on that step and the coordination of the resulting alcohol to the iron.

### Trimethylamine Coordination

**Scheme 3:**
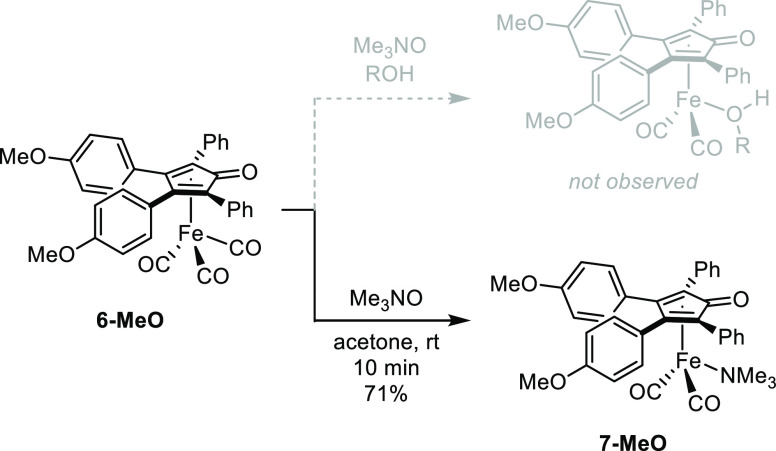
Attempted Formation of an Alcohol Adduct Led to Trimethylamine Compound **7-MeO**

(Cyclopentadienone)iron alcohol dicarbonyl compounds have been characterized by NMR spectroscopy and X-ray crystallography and are known to decompose quickly at room temperature.^8^ Our attempts to observe alcohol adducts of **6-MeO**, **6-Me**, and **6-H** by NMR spectroscopy under our hydrogenation and dehydrogenation reaction conditions were unsuccessful. Switching the solvent to benzene-*d*_6_ or toluene-*d*_8_ to eliminate competition with catalyst turnover still did not lead to an observable alcohol-iron complex. Instead, we observed peaks consistent with a trimethylamine-bound iron complex (**7-MeO**) that formed cleanly in acetone (Scheme 3). We proposed the formation of a similar trimethylamine-bound complex derived from **2** in acetone-*d*_6_ based on ^1^H and ^13^C NMR spectroscopy data while exploring the iron-catalyzed lactonization of diols,^21^ and Blondin, Kochem, and co-workers used both Mössbauer and ^13^C NMR spectroscopy to characterize the same complex.^53^ Recently, Bäckvall, and co-workers identified a (cyclopentadienone)iron-amine species as an intermediate in the dehydrogenation of amines to imines, which they characterized by X-ray crystallography.^54^ Decarbonylation of other iron carbonyl complexes with trimethylamine *N*-oxide has led to the formation of amine-bound species, although there was evidence for dimethylamine—not trimethylamine—coordination.^55^

Conveniently, **7-MeO** had limited solubility in acetone and was isolated by simple filtration. The air-stable, orange/brown solid was characterized by NMR spectroscopy (CDCl_3_) and high-resolution mass spectrometry, with diagnostic peaks for the trimethylamine methyl groups as a singlet at 2.24 ppm in the ^1^H NMR spectrum and at 56.8 ppm in the ^13^C{^1^H} NMR spectrum. Blondin, Kochem, and co-workers identified the same peak in the ^13^C{^1^H} NMR spectrum (CDCl_3_) of their NMe_3_ derivative of **2** at 58.2 ppm,^53^ which is consistent with our data.

**Figure 5:**
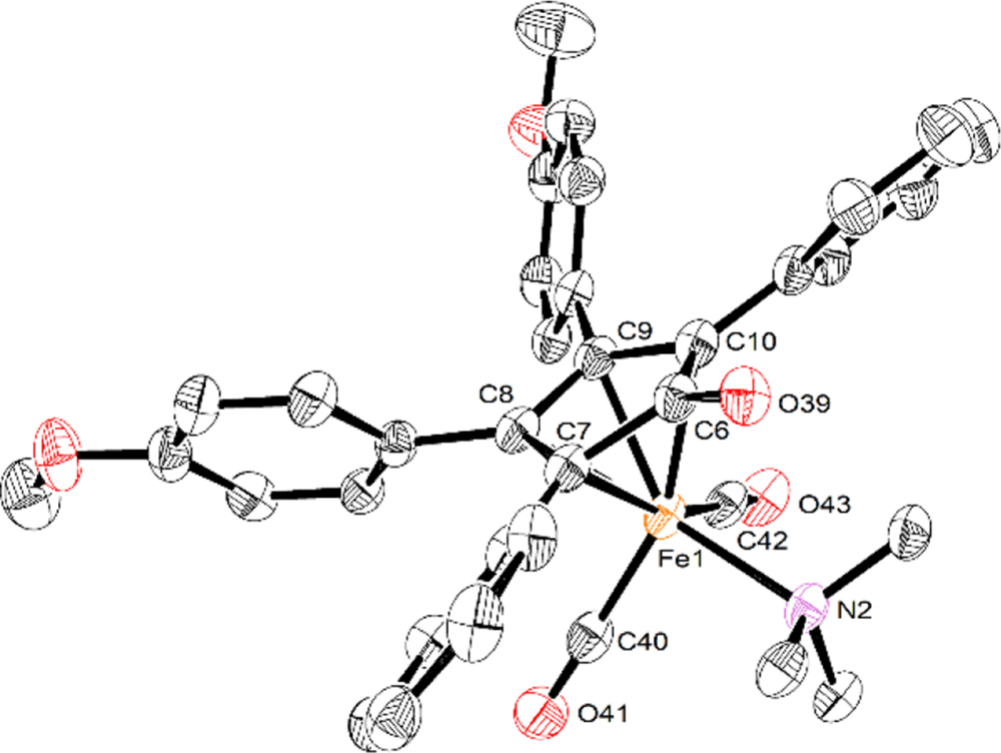
X-ray crystal structure of **7-MeO**, showing 50% probability ellipsoids. Hydrogen atoms have been omitted for clarity. Selected interatomic distances (Å): Fe(1)–N(2), 2.112(2); Fe(1)–C(6), 2.387(3); Fe(1)–C(7), 2.146(3); Fe(1)–C(8), 2.070(3); Fe(1)–C(9), 2.062(3); Fe(1)–C(10), 2.144(3); Fe(1)–C(40), 1.780(3); and Fe(1)–C(42), 1.773(3).

Additionally, we were able to characterize **7-MeO** by X-ray crystallography, and its solid-state structure is shown in Figure 5. There are similarities between the structure of **7-MeO** and **6-MeO** and a derivative of **7-MeO** where the trimethylamine ligand is replaced with a 1,3-dimethyl N-heterocyclic carbene (NHC).^44^ The four aromatic rings tilt out of the plane of the cyclopentadienone in all three complexes, and the average distances between the iron and the cyclopentadienone ring carbons—C(7)–C(10) in the structure of **7-MeO**—are approximately 0.25 Å shorter than the distance from the iron to the ketonic carbonyl carbon: Fe(1)–C(6) in **7-MeO**. As the third CO ligand is replaced with a strong donor and weaker π-acceptor (e.g., trimethylamine or an NHC), the remaining two Fe–CO bond distances—Fe(1)–C(40) and Fe(1)–C(42) in **7-MeO**—contract from an average of 1.81 Å in **6-MeO** to 1.78 Å in **7-MeO** to 1.76 Å in the NHC derivative of **7**. Knölker characterized a derivative of **2,** where two CO ligands were replaced by acetonitrile, and the average Fe–N bond distance was 1.941 Å,^56^ which is a little shorter than the Fe(1)–N(2) distance in **7-MeO**. The elongation of the bond may be due to the bulkier trimethylamine ligand compared to acetonitrile.

With the structure of **7-MeO** confirmed, we looked for evidence of the formation of trimethylamine-bound species under the reaction conditions. Trimethylamine *N*-oxide is commonly used to activate (cyclopentadienone)iron tricarbonyl compounds in solution, and the formation of **7-MeO** suggested that trimethylamine-bound species are likely to form under typical catalytic reaction conditions. We elected to use **6-Me** in our NMR studies because of the limited solubility of **7-MeO** in acetone-*d*_6_. When we treated a solution of **6-Me** in acetone-*d*_6_ with trimethylamine *N*-oxide, we observed a new signal at 7.93 ppm in the ^1^H NMR spectrum, which we assigned to **7-Me** (Supporting Information (SI), Figure S18). Conveniently, this aromatic signal was distinct from the most deshielded peak of **6-Me** at 7.64 ppm in acetone-*d*_6_ (SI, Figure S17), and it was consistent with how the most deshielded signal in **6-MeO** (7.53 ppm in CDCl_3_) shifted to 7.91 ppm in the ^1^H NMR spectrum of **7-MeO**. Additionally, the iron-ligated trimethylamine methyl protons appeared at 2.27 ppm. Trimethylamine adduct **7-Me** formed in less than 10 min at room temperature.

The equivalents of trimethylamine *N*-oxide added relative to **6-Me** affected the molar ratio of **6-Me** to **7-Me**. When one equivalent was used, the **6-Me**:**7-Me** ratio varied between 1:1 and 2:1. Very little **6-Me** was observed when 1.2–2 equiv of trimethylamine *N*-oxide was added; the major species was **7-Me**. These observations are consistent with those by Blondin, Kochem, and co-workers working with **2** in toluene and CDCl_3_.^53^

A transfer dehydrogenation of 2-heptanol was done in acetone-*d*_6_ using **6-Me** and trimethylamine *N*-oxide (1 equiv relative to **6-Me**), and both **6-Me** and **7-Me** were present as the major iron species in solution during catalysis as observed by ^1^H NMR spectroscopy (eq 1; SI, Figures S19 and S20). No evidence for an iron hydride (i.e., **C** in Scheme 1) or a diiron bridging hydride analogous to **3** was found down to −30 ppm.
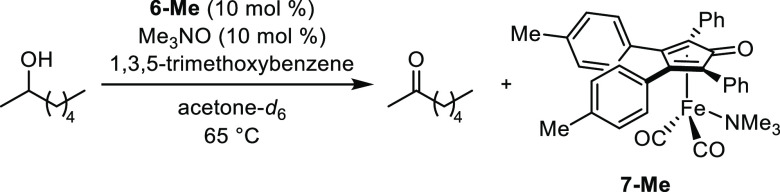
1

To determine whether the same iron species were present later in the reaction progress, a similar experiment was done using a 3:1 molar ratio of 2-heptanone to 2-heptanol to mimic the conditions at 75% conversion. An equimolar amount of isopropanol relative to 2-heptanone was added because it would be present as a byproduct. A ^1^H NMR spectrum of the solution at ambient temperature showed a very small peak at around −11 ppm, likely corresponding to an iron hydride (see below), but upon heating to 55 °C, it was not present and the only peaks corresponding to iron species matched those for **7-Me** (SI, Figures S21 and S22).

We performed a similar set of ^1^H NMR experiments in the presence of isopropanol. When 1 equiv of trimethylamine *N*-oxide was added to a solution of **6-Me** in 1:1 isopropanol/benzene-*d*_6_ at ambient temperature, two new peaks appeared downfield of the most deshielded signal for **6-Me** at 7.61 ppm: one at 7.73 ppm and one at 8.04 ppm (SI, Figure S24). The latter peak was assigned to **7-Me** based on its chemical shift relative to spectra of **7-Me** in CDCl_3_ and acetone-*d*_6_. Additionally, a small singlet at −10.72 ppm was present, which is consistent with the chemical shifts of (hydroxycyclopentadienyl)iron and ruthenium hydrides^6,9,12^ and was assigned to iron hydride **8-Me** (eq 2). We were unable to identify the structure of the compound that corresponded to the peak at 7.73 ppm, but integration values in the ^1^H NMR spectrum showed that the signal did not correlate to hydride **8-Me** and it was not free cyclopentadienone. The major iron species in solution were unreacted tricarbonyl compound **6-Me**, trimethylamine-bound **7-Me**, and the unidentified compound, and the ratio of **7-Me** to hydride **8-Me** was approximately 10:1. Upon heating to 65 °C, the **7-Me** to **8-Me** ratio shifted to 1:1.5, corresponding to an increase in the amount of iron hydride relative to the trimethylamine adduct at elevated temperatures (SI, Figure S25). We did not observe any other signals down to −30 ppm at rt or 65 °C, so it is unlikely that a diiron bridging hydride analogous to **3** formed.^57,58^

A transfer hydrogenation reaction of 2-butanone was monitored by ^1^H NMR spectroscopy using a 2:1 ratio of trimethylamine *N*-oxide to **6-Me** (eq 2; SI, Figure S26). The initial spectrum taken at 65 °C showed trimethylamine compound **7-Me** as the major species and the **7-Me** to hydride **8-Me** ratio was 4.7:1. No other iron hydrides were present. The use of excess trimethylamine *N*-oxide resulted in only small amounts of unreacted tricarbonyl **6-Me** and the same unidentified compound noted above. As the reaction continued to heat, a precipitate formed and peak broadening occurred in the spectrum, so no further reliable data was available. The same issue occurred when one or three equivalents of trimethylamine *N*-oxide were used relative to **6-Me**. Overall, these results are consistent with trimethylamine-bound species (**7**) forming in solution as a precatalyst under catalytic conditions.
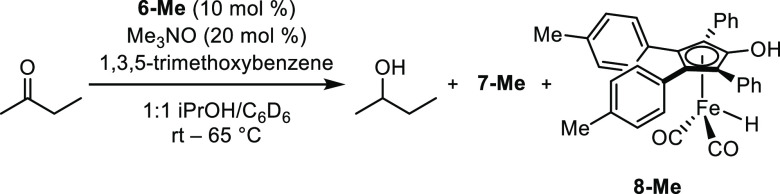
2

As with the transfer dehydrogenation reaction, we looked for iron species present in solution later in the reaction progress. A similar experiment to that shown in eq 2 was run using a 3:1 molar ratio of 2-butanol to 2-butanone (mimicking 75% conversion) with one equivalent of acetone added relative to 2-butanol. At both ambient temperature and 65 °C, the same iron compounds were present in the ^1^H NMR spectra as those described above: unreacted **6-Me**, **7-Me**, **8-Me**, and the unidentified species (SI, Figures S27 and S28).

### Catalytic Activity of Trimethylamine Adduct **7-MeO**

**Figure 6:**
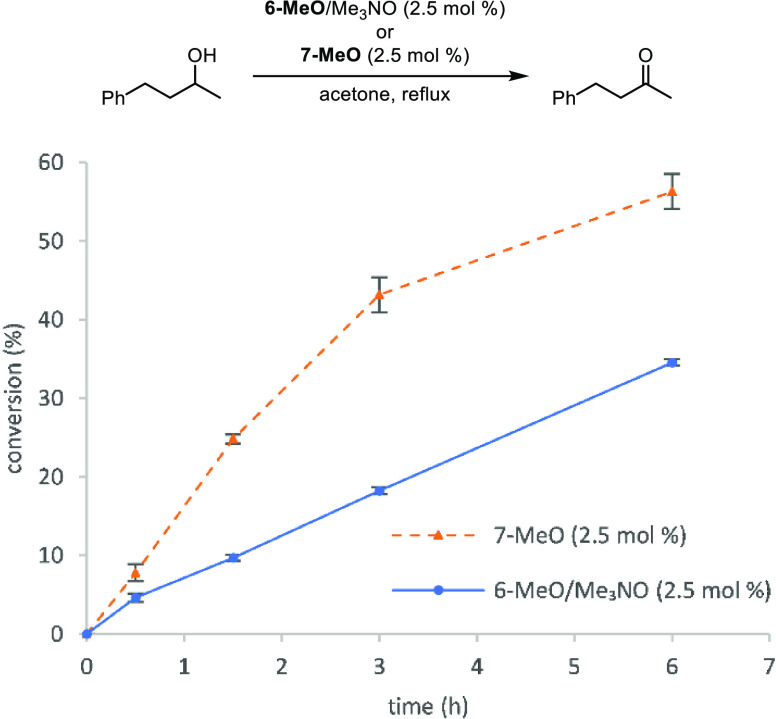
Conversion (%) vs time (h) for the transfer dehydrogenation of 4-phenyl-2-butanol using **6-MeO** + trimethylamine *N*-oxide (2.5 mol % each) or **7-MeO** (2.5 mol %). Plotted points are averages of at least two runs. Conversions were determined by GC relative to biphenyl.

(Cyclopentadienone)iron dicarbonyl compounds with labile ligands such as nitriles^59^ and phosphines^60^ are known to be catalytically active. Additionally, the analogous (tetraphenylcyclopentadienone)Ru(CO)_2_NMe_3_ complex has been prepared, and it reacted in ways that were consistent with a labile trimethylamine ligand.^61−63^ With the trimethylamine-bound **7-MeO** in hand, we could explore its activity directly without generating it in solution from the parent tricarbonyl compound. First, we compared the activity of **7-MeO** to **6-MeO** + trimethylamine *N*-oxide in the transfer dehydrogenation of 4-phenyl-2-butanol in acetone (Figure 6). Importantly, **7-MeO** was catalytically active. The initial reaction rate was faster with **7-MeO**, and after 24 h, the reaction reached 80% conversion. The difference in initial rates can be explained in conjunction with the NMR data, which showed that unactivated tricarbonyl compound (**6-Me**) and trimethylamine adduct (**7-Me**) are both present during transfer dehydrogenation when a 1:1 molar ratio of **6** and trimethylamine *N*-oxide are used as the catalyst system. There is less **7** present when it is generated in solution from **6** compared to when it is added directly, which results in a slower initial rate for the reaction catalyzed by **6-MeO** + trimethylamine *N*-oxide. These data also support **7-MeO** as the “active precatalyst” for the reaction.

**Figure 7:**
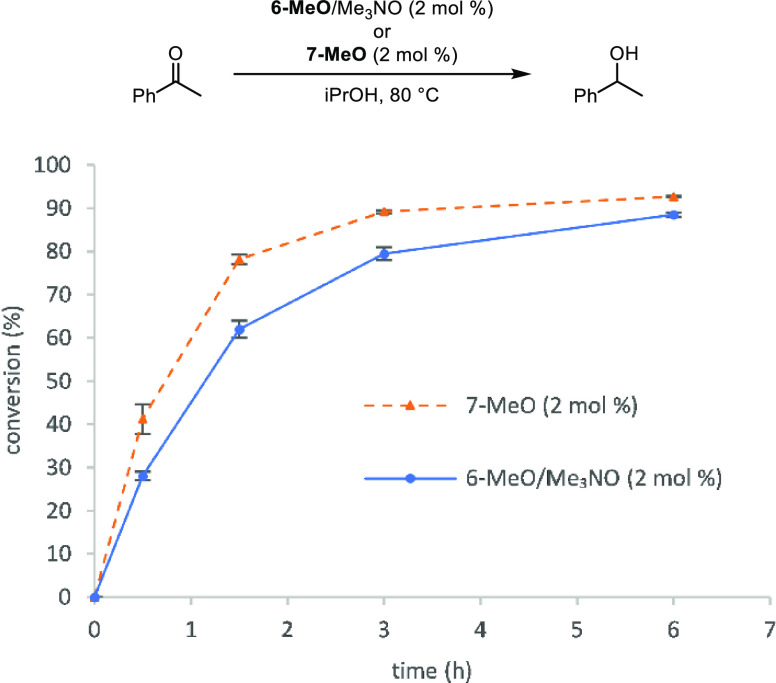
Conversion (%) vs time (h) for the transfer hydrogenation of acetophenone using **6-MeO** + trimethylamine *N*-oxide (2 mol % each) or **7-MeO** (2 mol %). Plotted points are averages of at least two runs. Conversions were determined by GC relative to biphenyl.

Compound **7-MeO** was also active in the transfer hydrogenation of acetophenone in isopropanol (Figure 7). Like the dehydrogenation above, the initial reaction rate was higher with **7-MeO** than with **6-MeO** + trimethylamine *N*-oxide and higher conversions were obtained within 6 h compared to dehydrogenations (Figure 6). From the ^1^H NMR spectroscopic studies, we know that a mixture of iron species is present in solution when **6-Me** is treated with one equivalent of trimethylamine *N*-oxide, including catalytically unreactive **6-Me**. When **7-MeO** is used, presumably all of the iron species in solution are catalytically active, which explains the faster initial rate.

As proposed in Scheme 1, a coordination site on iron must be available (i.e., unsaturated species **B** must form) for catalytic turnover to occur. Therefore, trimethylamine should dissociate from **7-MeO** to form **B** and provide entry into the catalytic cycle. If the pre-equilibrium between **7-MeO** and **B** favors **7-MeO**, it should limit the catalyst turnover. To test this hypothesis, the same reactions shown in Figures 6 and 7 were run under an atmosphere of trimethylamine, which dramatically reduced reaction rates and conversions (SI, Figures S1 and S2). After 6 h, the conversion of the transfer dehydrogenation of 4-phenyl-2-butanol was at less than 2% and the transfer hydrogenation of acetophenone using **7-MeO** reached less than 9% conversion. Excess trimethylamine inhibited catalyst turnover, which is consistent with the requirement that unsaturated species **B** must form in a pre-equilibrium.

### Kinetic Isotope Effects

To gain insight into the turnover-limiting step, acetophenone was reduced in a transfer hydrogenation reaction using either **6-MeO** + trimethylamine *N*-oxide (2 mol % each) or **7-MeO** (2 mol %), with either isopropanol or isopropanol-*d*_8_ as the solvent and hydrogen source. The reactions were run multiple times in parallel, and their initial rates were compared. For the **6-MeO** + trimethylamine *N*-oxide-catalyzed process, *k*_H_/*k*_D_ = 3.66 ± 0.14 and *k*_H_/*k*_D_ = 3.76 ± 0.11 when **7-MeO** was used. These values are equal within error and are consistent with a primary kinetic isotope effect, indicating that hydrogen transfer is taking place during the slow step of the reaction. Casey and co-workers determined a similar value (*k*_H_/*k*_D_ = 3.6 ± 0.3) when they reduced benzaldehyde with a ruthenium derivative of **8-Me**,^12^ and the Williams group studied the reverse reaction—the oxidation of benzyl alcohol (PhC(D)HOH)—using **6-H** and found an internal competition isotope effect of *k*_H_/*k*_D_ = 3.6 ± 0.9.^64^

**Figure 8:**
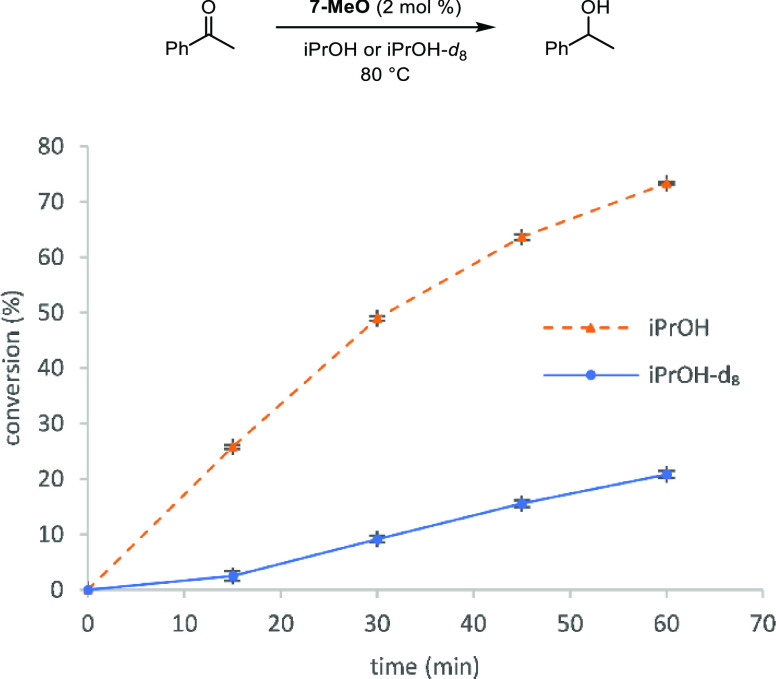
Conversion (%) vs time (h) for the transfer hydrogenation of acetophenone in isopropanol and isopropanol-*d*_8_, indicating an induction period in the reaction in isopropanol-*d*_8_. Plotted points are the averages of at least two runs. Conversions were determined by GC relative to biphenyl.

Throughout our kinetic studies with (cyclopentadienone)iron carbonyl compounds, we never observed an induction period until we used isopropanol-*d*_8_ as the solvent and hydrogen source. In acetophenone transfer hydrogenations with both **6-MeO** + trimethylamine *N*-oxide and **7-MeO** in isopropanol-*d*_8_, the reaction rates during the first 15 min were slower than that during the subsequent 45 min. Figure 8 compares the initial progress for acetophenone hydrogenations using **7-MeO**, and the induction period was observed in isopropanol-*d*_8_ but not in isopropanol. Because the induction period occurred in the reaction with trimethylamine adduct **7-MeO**, it was not caused by the activation of tricarbonyl compound **6-MeO** with trimethylamine *N*-oxide. Instead, we attribute it to the formation of the active reducing agent, iron hydride **8-MeO**, which forms in solution during transfer hydrogenations based on our NMR studies using **6-Me**. Hydride **8-MeO** must form quickly enough in isopropanol that no induction period is observed, but in isopropanol-*d*_8_, iron deuteride takes more time to build up in solution. The initial rates used to determine *k*_D_ were measured after the induction periods ended.

### Effect of Substrate Electronics

**Figure 9:**
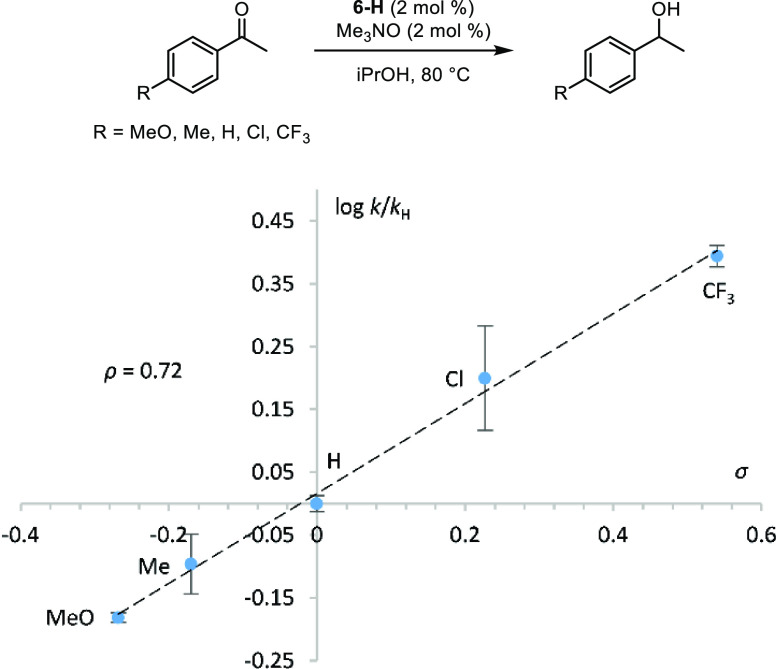
Hammett plot (log[*k*/*k*_H_] vs σ) for the transfer hydrogenation of 4′-substituted acetophenones using catalyst **6-H**.

The kinetic isotope effect experiments for transfer hydrogenations were consistent with hydrogen transfer taking place during the turnover-limiting step of the catalytic cycle, which can occur at two different points: transfer from isopropyl alcohol to unsaturated iron species **B** or from iron hydride **C** to the carbonyl compound (Scheme 1). Electron density on the catalyst decreased during the slow step, according to the Hammett plot in Figure 3, which was consistent with the latter option. To provide further support for a turnover-limiting step involving transfer of hydrogen from the iron catalyst to the carbonyl compound, 4′-substituted acetophenones were reduced using **6-H** and trimethylamine *N*-oxide under our usual conditions, and their initial rates were measured and compared to the substituents’ sigma values (Figure 9).^65^ Substrate electronics affected the reaction rates, which is consistent with the reaction of the ketone occurring in the turnover-limiting step of the catalytic cycle. Additionally, the positive reaction constant (ρ = 0.72) corresponds to increased electron density on the substrate in the transition state, which is expected when the substrate is being reduced and the catalyst is donating a hydride. A larger but also a positive ρ value of 1.77 ± 0.08 was found when para-substituted benzaldehydes were reduced with a ruthenium derivative of **8-Me** where one CO ligand was replaced with triphenylphosphine.^66^

**Figure 10:**
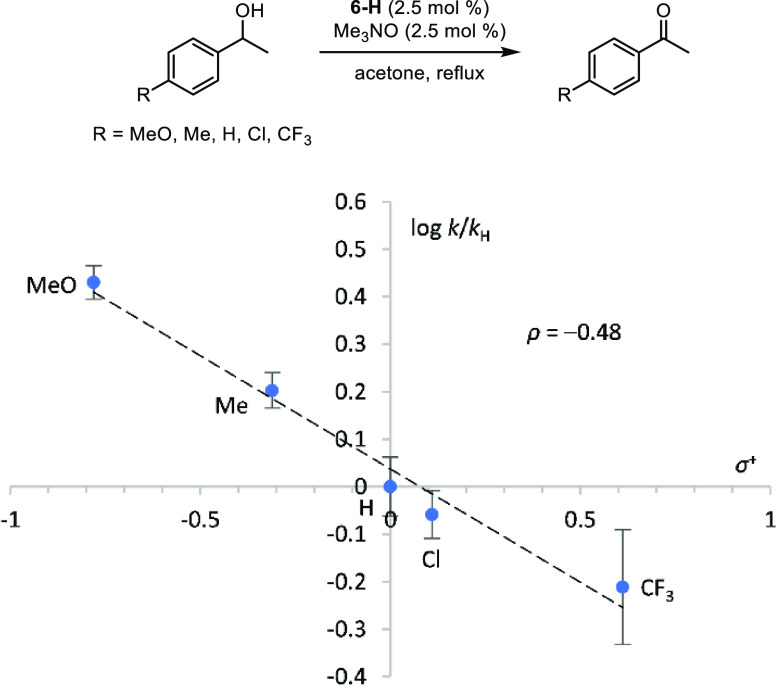
Hammett plot (log[*k*/*k*_H_] vs σ^+^) for the transfer dehydrogenation of *p*-substituted 1-phenylethanols using catalyst **6-H**.

Transfer dehydrogenations of *p*-substituted 1-phenylethanols with **6-H** and trimethylamine *N*-oxide in acetone were also carried out, and the initial reaction rates varied, indicating that the alcohol was involved in the turnover-limiting step (Figure 10). Additionally, the Hammett plot fit was significantly better using σ^+^ values instead of σ values.^67^ The combination of a negative reaction constant and a good fit with σ^+^ values indicates a positive charge building up on the benzylic carbon in the transition state, which is consistent with hydride transfer from the alcohol α-carbon to the iron center. A similar trend resulting in similar conclusions was observed with Shvo’s catalyst (**3**), although a larger ρ value (−0.89 ± 0.05) was obtained when σ values were used.^64^

### Proposed Catalytic Cycles

**Scheme 4:**
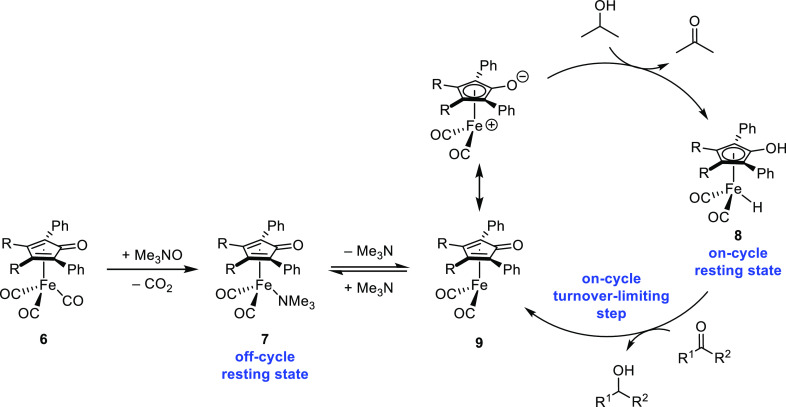
Proposed Catalytic Cycle for Transfer Hydrogenations

Taken together, these results provide insights into the catalytic cycles of transfer hydrogenations and dehydrogenations using (cyclopentadienone)iron tricarbonyl compounds activated with trimethylamine *N*-oxide. The proposed transfer hydrogenation cycle is shown in Scheme 4. When tricarbonyl compound **6** is activated with trimethylamine *N*-oxide, carbon dioxide is lost and trimethylamine-ligated **7** forms quickly with no visible induction period. In addition to being isolated and characterized by NMR spectroscopy, mass spectrometry, and X-ray crystallography, **7** was observed by NMR spectroscopy under reducing conditions that corresponded to both low and high conversions of ketone to alcohol and was an active precatalyst for transfer hydrogenations. Trimethylamine dissociates, and the unsaturated species **9** reacts with isopropanol to form iron hydride **8**. Excess trimethylamine inhibits the formation of **9** and catalyst turnover. Compound **8** was also observed by NMR spectroscopy at both low and high conversions of ketone to alcohol and serves as the catalyst resting state in the catalytic cycle. While no induction period was observed for the generation of **8** from **7** in isopropanol, one was observed in isopropanol-*d*_8_.

Finally, a collection of data supports the transfer of hydrogen from **8** to the carbonyl compound as the turnover-limiting step. Electron-rich catalysts were more active, and the Hammett plot in Figure 3 illustrates that electron density on the catalyst decreased during the slow step. Kinetic isotope experiments showed that hydrogen transfer occurred during the turnover-limiting step, and the data in Figure 9 illustrate that the ketone gains electron density as it reacts in that step. The large excess of isopropanol drives the reduction reaction forward, but each step in the cycle is reversible.

Our proposed catalytic cycle for transfer hydrogenations is similar to those proposed for carbonyl reductions with H_2_,^8,10,11^ although dihydrogen activation is not required. Bütikofer and Chen prepared and studied the carbonyl reduction mechanism of an anionic, water-soluble derivative of **2** using mass spectrometry, and they provided evidence for an unsaturated iron species analogous to **9** as the resting state in their catalytic cycle,^68^ which differs from our proposal of trimethylamine-bound **7** and iron hydride **8** as the primary catalyst resting states. They used a dicarbonyl acetonitrile catalyst that was not activated by trimethylamine *N*-oxide, so a compound analogous to **7** would be unable to form. When we monitored transfer hydrogenations by ^1^H NMR spectroscopy, we had an unidentified signal at 7.73 ppm that could be due to **9** or a dimer of **9**,^30,69^ but it was present in small amounts when the sample was heated with 2-butanone. They propose acetophenone reduction as their turnover-limiting step, which matches our conclusion. While we were able to observe hydride **8** spectroscopically under transfer hydrogenation conditions, Tamm and co-workers did not see a similar iron hydride when they treated a (tetraaminocyclopentadienone)iron dicarbonyl acetonitrile compound under the same conditions.^51^ The difference could be due to their use of an acetonitrile-ligated iron compound, or it could indicate a different catalyst resting state—and a different turnover-limiting step—when catalysts bearing highly electron-rich, amine-substituted cyclopentadienones are used.

**Scheme 5:**
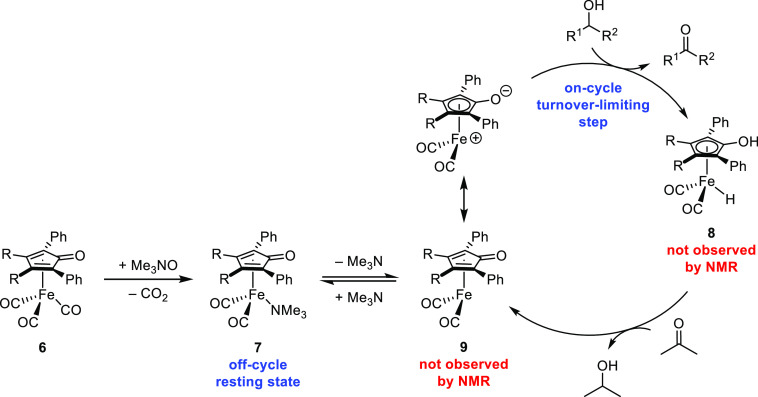
Proposed Catalytic Cycle for Transfer Dehydrogenations

The proposed transfer dehydrogenation cycle is shown in Scheme 5. While the initial rate data was not clean using catalysts **6**, electron-rich catalysts were still favored and reacted more quickly than electron-poor catalysts. If the turnover-limiting step is hydrogen transfer from the alcohol to the catalyst, the same justification used for derivatives of Shvo’s catalyst (**3**) could apply—electron-rich catalysts react faster by increasing the basicity of the cyclopentadienone carbonyl oxygen, leading to a stronger hydrogen bond with the incoming alcohol and decreasing the energy of hydrogen transfer to the catalyst.^47^ The only iron species observed by ^1^H NMR spectroscopy during catalysis—at both low and high conversions of alcohol to ketone—were unactivated tricarbonyl **6** and trimethylamine-ligated **7**, neither of which are on-cycle intermediates, and the resting state of the catalyst is likely off-cycle **7**. The reaction is favored by electron-donating groups on the alcohol, which is consistent with alcohol dehydrogenation via hydride transfer occurring in the turnover-limiting step. Each step in the dehydrogenation catalytic cycle is also reversible, and excess trimethylamine inhibits catalyst turnover.

The Williams group studied the reactivity of **6-H**—activated by water, not trimethylamine-*N*-oxide—in the oxidation of 2° benzylic alcohols and concluded that C–H bond cleavage is electrophilic and occurs during the turnover-limiting step in the cycle, which is consistent with our results.^64^ Similar conclusions were drawn when Shvo’s catalyst (**3**) was used in alcohol dehydrogenations.^13,64^ Guan and co-workers reacted crude iron hydride **8-Me** with acetone in toluene-*d*_8_ and observed multiple iron species by ^1^H NMR spectroscopy, including what appeared to be a diiron bridging hydride at −22.7 ppm similar in structure to Shvo’s catalyst (**3**).^58^ When heated, **3** dissociates into two species: ruthenium analogues of unsaturated species **9-H** and monomeric hydride **8-H**. We did not observe any monomeric or bridging iron hydrides in our transfer dehydrogenation ^1^H NMR spectroscopic studies, which were done in excess acetone-*d*_6_. Additionally, Guan’s group did not use trimethylamine-*N*-oxide to activate the catalyst; therefore, no trimethylamine was present. If a diiron bridging hydride reversibly formed in our reactions, trimethylamine would bind to monomeric unsaturated **9** to form trimethylamine-bound **7**, leaving hydride **8** available to rapidly react with excess acetone.

Our proposed turnover-limiting step for dehydrogenations is not the same as that for hydrogenations, which could be caused by the use of different solvents. In hydrogenations, there is a large excess of isopropyl alcohol (i.e., the hydrogen donor), whereas acetone—the hydrogen acceptor/terminal oxidant—is in excess in dehydrogenations. These solvent concentration differences affect the iron species in solution—iron hydride **8** was observed in isopropanol but not in acetone—and shift the turnover-limiting step in the catalytic cycle to either hydrogen delivery to the ketone in hydrogenations or hydrogen removal from the alcohol in dehydrogenations. For a given reaction, the same iron species are observed in solution by ^1^H NMR spectroscopy at both low and high conversions, which supports the high concentration of the solvent (i.e., hydrogen acceptor and donor) as the primary factor affecting on-cycle catalyst resting states.

## Conclusions

A series of (tetraarylcyclopentadienone)iron tricarbonyl compounds with electron-donating and -withdrawing substituents were prepared, and their activities in transfer hydrogenations and dehydrogenations were explored. Catalysts with electron-rich cyclopentadienone ligands reacted more quickly than those without in both transfer hydrogenations and dehydrogenations. A trimethylamine-ligated (cyclopentadienone)iron dicarbonyl compound was isolated and characterized by NMR spectroscopy, mass spectrometry, and X-ray crystallography. It catalyzed both hydrogenation and dehydrogenation reactions and was present in solution under both reaction conditions when the tricarbonyl precursor was treated with trimethylamine *N*-oxide. The mechanisms of these hydrogen transfer processes were explored through kinetic isotope experiments—which provided evidence that hydrogen transfer occurred in the turnover-limiting step of the catalytic cycle for transfer hydrogenations—and by modifying substrate electronics.

Ultimately, catalytic cycles for transfer hydrogenations and dehydrogenations were proposed. For hydrogenations, we propose two catalyst resting states: an off-cycle trimethylamine adduct and an on-cycle iron hydride. The turnover-limiting step is the transfer of hydrogen to the carbonyl substrate from the iron hydride. For dehydrogenations, we propose the trimethylamine adduct as the primary catalyst resting state, and the turnover-limiting step is the transfer of hydrogen from the alcohol substrate to the catalyst. These mechanistic insights can be used to design more active and/or longer-lived catalysts for transfer hydrogenations of carbonyl compounds and transfer dehydrogenations of alcohols.

## Experimental Section

### General Information

All reactions were performed under an atmosphere of nitrogen unless otherwise noted. Commercial chemicals were used as received. Cyclopentadienones **5-MeO**(70) and **5-Me**,^71^ iron compounds **6-MeO**,^44^**6-Me**,^58^ and **6-H**,^40^ and 1,4-dimethylpiperazine-2,3-dione (DMPD)^45^ were prepared according to the published procedures. Reagent-grade acetone and isopropanol (for the transfer hydrogenations and dehydrogenations) were degassed by bubbling N_2_ through them for at least 15 min prior to use, but no attempt was made to remove residual water. All ^1^H and ^13^C{^1^H} NMR spectra were recorded at ambient temperature at 400 and 100 MHz, respectively, on a Bruker Avance Neo 400 MHz FT-NMR spectrometer unless otherwise noted. Chemical shifts are reported in parts per million (ppm) relative to tetramethylsilane (TMS) for spectra taken in CDCl_3_. ^1^H NMR spectra taken in acetone-*d*_6_ and benzene-*d*_6_ used the residual solvent peaks, 2.05 and 7.16 ppm, respectively, as references. High-resolution mass spectrometry data were collected at the Johns Hopkins University Mass Spectrometry Facility. Analytical thin-layer chromatography (TLC) was performed using silica gel 60 F254 precoated plates (0.25 mm thickness) with a fluorescent indicator. Flash column chromatography was performed using silica gel 60 (230–400 mesh). Gas chromatograms were collected on a Thermo Scientific Trace 1300 gas chromatograph with an AI 1310 autosampler and an FID. A TR-5 (5% phenyl methylpolysiloxane) column (30 m length × 0.25 mm ID × 0.25 μm film thickness) was used under the following method conditions: 110 °C for 5 min, ramp 20 °C/min to 250 °C, and hold at 250 °C for 2 min. The carrier gas was helium, used at a constant flow rate of 1 mL/min. A sample volume of 1 μL was added to the 300 °C injector at a 30:1 split ratio, and the FID temperature was 250 °C. Retention times (4.7 min for acetophenone, 7.6 min for 4-phenyl-2-butanol, 4.5 min for 1-phenylethanol, and 9.0 min for biphenyl) were determined using pure samples.

### Catalyst Synthesis

#### 4,4′-Dichlorobenzil (**4-Cl**)^72^

A solution of 1.6 M *n*-butyllithium in hexanes (8.8 mL, 14.1 mmol) was added dropwise to a solution of 4-bromochlorobenzene (2.83 g, 14.8 mmol) in anhydrous THF (12 mL) at −78 °C. After 30 min at −78 °C, the pale green solution was transferred by cannula to a solution of DMPD (1.00 g, 7.03 mmol) in anhydrous THF (12 mL) at rt and stirred for 1 h. The pale-yellow solution was diluted with 50 mL of dichloromethane and washed with 50 mL of 10% aqueous HCl, 50 mL of brine, dried over anhydrous sodium sulfate, and evaporated under reduced pressure. The resulting solid was triturated in hexanes at 4 °C to afford 1.71 g (6.12 mmol, 87%) of **4-Cl** as yellow crystals. ^1^H NMR (CDCl_3_, 400 MHz): δ 7.94–7.90 (m, 4H), 7.52–7.49 (m, 4H). ^13^C{^1^H} NMR (CDCl_3_, 100 MHz): δ 192.4, 141.8, 131.3, 131.1, 129.5.

#### 4,4′-Bis(trifluoromethyl)benzil (**4-CF_3_**)^73^

A solution of 1.6 M *n*-butyllithium in hexanes (13.8 mL, 22.1 mmol) was added dropwise over 5 min to a solution of 4-bromobenzotrifluoride (5.22 g, 23.2 mmol) in 23 mL of anhydrous THF at −78 °C. The pale green solution was immediately transferred by cannula to a suspension of DMPD (1.50 g, 10.6 mmol) in 22 mL of anhydrous THF at rt. (Note: holding the 4-lithiobenzotrifluoride solution for 30–60 min at −78 °C before the transfer to DMPD resulted in decreased yields.) After 2 h, the light orange solution was diluted with 75 mL of dichloromethane, washed with 10% aqueous HCl, 75 mL of brine, dried over sodium sulfate, and evaporated under reduced pressure. The resulting solid was triturated with 10 mL of hexanes at 4 °C to afford 2.52 g (7.28 mmol, 69%) of **4-CF**_**3**_ as a yellow solid. ^1^H NMR (CDCl_3_, 400 MHz): δ 8.12 (d, 4H, *J* = 8.4 Hz), 7.81 (d, 4H, *J* = 8.4 Hz). ^13^C{^1^H} NMR (CDCl_3_, 100 MHz): δ 191.9, 136.2 (q, *J*_CF_ = 32.7 Hz), 135.2, 130.3, 126.2 (q, *J*_CF_ = 3.6 Hz), 123.3 (q, *J*_CF_ = 271.3).

#### 3,4-Bis(4-chlorophenyl)-2,5-diphenylcyclopenta-2,4-dien-1-one (**5-Cl**)^74^

To a solution of **4-Cl** (2.55 g, 9.12 mmol) and 1,3-diphenylacetone (1.92 g, 9.12 mmol) in 14 mL of 95% ethanol at 80 °C was added 1.4 mL (4.98 mmol) of 3.56 M KOH in 95% ethanol, and the solution became dark red. After 45 min at 80 °C, the solution was cooled to 4 °C, and the solid was collected by vacuum filtration and washed with 95% ethanol at 4 °C to afford 3.47 g (7.66 mmol, 84%) of **5-Cl** as dark purple crystals. ^1^H NMR (CDCl_3_, 400 MHz): δ 7.27–7.24 (m, 6H), 7.21–7.17 (m, 8H), 6.87–6.84 (m, 4H). ^13^C{^1^H} NMR (CDCl_3_, 100 MHz): δ 199.6, 152.6, 134.8, 131.2, 130.8, 130.3, 130.1, 128.6, 128.3, 127.9, 125.9.

#### 2,5-Diphenyl-3,4-bis(4-(trifluoromethyl)phenyl)cyclopenta-2,4-dien-1-one (**5-CF**_**3**_)

To a solution of **4-CF**_**3**_ (0.500 g, 1.45 mmol) and 1,3-diphenylacetone (0.324 g, 1.54 mmol) in 2.2 mL of 100% ethanol at 75 °C was added 0.22 mL (0.77 mmol) of 3.5 M KOH in 100% ethanol. The dark purple solution stirred at reflux for 30 min was cooled to 4 °C, and the solid was collected by vacuum filtration and washed with 100% ethanol at 4 °C to afford 0.397 g (0.76 mmol, 53%) of **5-CF**_**3**_ as purple crystals. ^1^H NMR (CDCl_3_, 400 MHz): δ 7.47 (d, 4H, *J* = 8.0 Hz), 7.28–7.26 (m, 6H), 7.201–7.17 (m, 4H), 7.05 (d, 4H, *J* = 8.0 Hz). ^13^C{^1^H} NMR (CDCl_3_, 100 MHz): δ 199.4, 152.2, 136.5, 130.8 (q, *J*_CF_ = 32.6 Hz), 130.1, 129.8, 129.6, 128. 4, 128.2, 126.7, 125.3 (q, *J*_CF_ = 3.7 Hz), and 123.8 (q, *J*_CF_ = 270.7 Hz). HRMS (FAB) for C_31_H_18_F_6_O: calculated for [M]^+^*m*/*z* = 520.1262, found *m*/*z* = 520.12683.

#### [2,5-Diphenyl-3,4-bis(4-chlorophenyl)cyclopentadienone]iron Tricarbonyl (**6-Cl**)^75^

A solution of **5-Cl** (1.00 g, 2.21 mmol) and diiron nonacarbonyl (0.802 g, 2.21 mmol) in 10 mL of toluene was heated to 110 °C for 18 h in a sealed, thick-walled flask. The reaction mixture was filtered through Celite, the volatiles were removed under reduced pressure, and the crude product was purified by flash chromatography (85% hexanes, 15% ethyl acetate). The resulting solid was triturated in 4 °C hexanes and collected by vacuum filtration to afford 723 mg (1.24 mmol, 55%) of **6-Cl** as a yellow solid. ^1^H NMR (CDCl_3_, 400 MHz): δ 7.52–7.50 (m, 4H), 7.28–7.25 (m, 6H), 7.17 (d, *J* = 8.4 Hz, 4H), 7.06 (d, *J* = 8.4 Hz, 4H). ^13^C{^1^H} NMR (CDCl_3_, 100 MHz): δ 208.2, 169.7, 135.1, 133.0, 130.3, 128.6, 128.4, 128.3, 128.2, 102.5, 82.7. HRMS (FAB) for C_32_H_18_Cl_2_FeO_4_: calculated for [M + H]^+^*m*/*z* = 593.00098, found *m*/*z* = 593.00135.

#### [2,5-Diphenyl-3,4-bis(4-(trifluoromethyl)phenyl)cyclopentadienone]iron Tricarbonyl (**6-CF**_**3**_)

A solution of **5-CF**_**3**_ (0.306 g, 0.59 mmol) and iron pentacarbonyl (0.15 mL, 1.5 mmol) in 5 mL of toluene was heated to 140 °C for 24 h in a sealed, thick-walled flask. The volatiles were removed under reduced pressure, and the crude product was purified by flash chromatography (98% CH_2_Cl_2_, 2% methyl *t*-butyl ether) to afford 203 mg (0.307 mmol, 52%) of **6-CF**_**3**_ as a yellow solid. ^1^H NMR (CDCl_3_, 400 MHz): δ 7.51–7.49 (m, 4H), 7.45 (d, *J* = 8.0 Hz, 4H), 7.28–7.25 (m, 10H). ^13^C{^1^H} NMR (CDCl_3_, 100 MHz): δ 208.0, 169.7, 133.9, 132.1, 131.1 (q, *J*_CF_ = 32.8 Hz), 130.2, 130.0, 128.4, 125.3 (q, *J*_CF_ = 3.6 Hz), 123.6 (q, *J*_CF_ = 270.8 Hz), 102.2, 82.8. HRMS (FAB) for C_34_H_18_F_6_FeO_4_: calculated for [M + H]^+^*m*/*z* = 661.05370, found *m*/*z* = 661.05362.

#### [2,5-Diphenyl-3,4-bis(4-methoxyphenyl)cyclopentadienone]iron Dicarbonyl trimethylamine (**7-MeO**)

A solution of **6-MeO** (200 mg, 0.342 mmol) and anhydrous trimethylamine *N*-oxide (27 mg, 0.36 mmol) in 1.7 mL of acetone was stirred at rt for 1h. The light brown precipitate was collected by vacuum filtration and washed with 4 °C hexanes to afford 150 mg (0.243 mmol, 71%) of **7-MeO**. ^1^H NMR (CDCl_3_, 400 MHz): δ 7.91 (d, 4H, *J* = 6.8 Hz), 7.20–7.14 (m, 6H), 7.04 (d, 4H, *J* = 8.8 Hz), 6.61 (d, 4H, *J* = 8.4 Hz), 3.69 (s, 6H), 2.24 (s, 9H). ^13^C{^1^H} NMR (CDCl_3_, 100 MHz): δ 215.3, 164.1, 158.7, 134.1, 132.8, 130.0, 127.7, 126.2, 123.7, 112.9, 98.8, 81.4, 56.8, 55.0. HRMS (FAB) for C_36_H_33_FeNO_5_: calculated for [M + H]^+^*m*/*z* = 616.17864, found *m*/*z* = 616.17894.

### X-ray Crystallography

X-ray quality crystals of **7-MeO** were grown by vapor diffusion of hexanes into a dichloromethane solution. Diffraction data were collected on an Oxford Diffraction Gemini-R diffractometer, using Mo–Kα radiation at 110 K. A crystal was mounted on a Hampton Research Cryoloop using Paratone-N oil. Unit cell determination, data collection and reduction, and empirical absorption correction were performed using the CrysAlisPro software package^76^ Direct method structure solution was accomplished using SIR92,^77^ and full-matrix least-squares refinement was carried out using CRYSTALS.^78^ All non-hydrogen atoms were refined anisotropically, hydrogen atoms were placed in calculated positions, and their positions were initially refined using distance and angle restraints. All hydrogen positions were fixed in place for the final refinement cycles. Two molecules are present in the asymmetric unit, related by a noncrystallographic pseudoglide plane along the *b* axis. Highly disordered solvent was present in the unit cell; correction for this residual density was performed using the option SQUEEZE in the program package PLATON.^79^ A total of 36 electrons per unit cell were removed from a total potential solvent-accessible void of 589.6 Å^3^. We note that the void volume is sufficient to include approximately three hexane molecules or six dichloromethane molecules per unit cell and that either case (or a combination of solvents) would involve significantly more than 36 electrons per unit cell. It is likely that solvent escaped from the crystal through evaporation prior to X-ray data collection.

#### Kinetic Experiments

Note: If too many samples were removed from a reaction mixture, the final conversion after 24 h was lower than the 24 h conversion from the same reaction mixture with fewer samples taken. Increasing the total reaction volume (while holding the reactant/reagent concentrations constant) led to matching conversions.

##### Transfer hydrogenations Monitored over <24 h

A solution of acetophenone (300 mg, 2.5 mmol, 1 equiv) and biphenyl (96 mg, 0.63 mmol, 0.25 equiv) was prepared in 4 mL of isopropanol or isopropanol-*d*_8_. A 25 μL aliquot was removed, diluted with 1 mL of acetone, and analyzed by gas chromatography to give the *t* = 0 chromatogram. When the reaction was done under an atmosphere of trimethylamine, trimethylamine gas was bubbled through the solution for 60 s and a balloon of trimethylamine was placed atop the condenser. Iron catalyst **6** (0.05 mmol, 2 mol %) or **7-MeO** (31 mg, 0.05 mmol, 2 mol %) was added to the reaction solution, which was placed in an 80 °C oil bath. When **6** was used, a solution of anhydrous trimethylamine *N*-oxide (3.8 mg, 0.05 mmol, 2 mol %) in 1 mL of isopropanol or isopropanol-*d*_8_ was added to the reaction solution. When **7-MeO** was used, no trimethylamine *N*-oxide was added and 1 mL of isopropanol or isopropanol-*d*_8_ was used to bring the volume to 5 mL. Aliquots (200 μL) of the solution were removed at desired times and diluted with 1 mL of hexanes. Residual iron was removed from each aliquot by adding it to a Pasteur pipet half filled with silica gel and eluting with 4 mL of 1:1 hexanes/ethyl acetate. A 1.2 mL sample of the eluted solution was analyzed by gas chromatography. Conversion was determined based on how much acetophenone had been consumed compared to the amount of acetophenone in the *t* = 0 chromatogram relative to the internal standard (biphenyl). Each reaction was run at least twice, and the average conversions were reported. The error was calculated as either the difference from the actual value to the average (when two runs were compared) or one standard deviation (when more than two runs).

##### Transfer hydrogenations Monitored over >24 h

A solution of acetophenone/substituted acetophenone (10 mmol, 1 equiv) and biphenyl (386 mg, 2.5 mmol, 0.25 equiv) was prepared in 20 mL of isopropanol. A 50 μL aliquot was removed, diluted with 1 mL of acetone, and analyzed by gas chromatography to give a *t* = 0 chromatogram. Iron catalyst **6** (0.2 mmol, 2 mol %) and anhydrous trimethylamine *N*-oxide (15 mg, 0.2 mmol, 2 mol %) were added to the reaction solution, which was placed in an 80 °C oil bath. Aliquots (200 μL) of the solution were removed at the desired times and diluted with 1 mL hexanes. Residual iron was removed from each aliquot by adding it to a Pasteur pipet half filled with silica gel and eluting with 4 mL 1:1 hexanes/ethyl acetate. A 1.2 mL sample of the eluted solution was analyzed by gas chromatography. Conversion was determined based on how much reactant had been consumed compared to the amount of reactant in the *t* = 0 chromatogram relative to the internal standard (biphenyl). Each reaction was run at least twice, and the average conversions were reported. The error was calculated as either the difference from the actual value to the average (when comparing two runs) or one standard deviation (when comparing more than two runs).

##### Transfer Dehydrogenations

A solution of 4-phenyl-2-butanol (1.50 g, 10 mmol, 1 equiv) or *p-*substituted 1-phenylethanol (10 mmol, 1 equiv) and biphenyl (386 mg, 2.5 mmol, 0.25 equiv) was prepared in 20 mL acetone. A 50 μL aliquot was removed, diluted with 1 mL of acetone, and analyzed by gas chromatography to give the *t* = 0 chromatogram. Iron catalyst **6** (0.25 mmol, 2.5 mol %) and anhydrous trimethylamine *N*-oxide (19 mg, 0.25 mmol, 2.5 mol %) were added to the reaction solution, which was placed in a 60 °C oil bath. Aliquots (200 μL) of the solution were removed at the desired times and diluted with 1 mL hexanes. Residual iron was removed from each aliquot by adding it to a Pasteur pipet half filled with silica gel and eluting with 4 mL 1:1 hexanes/ethyl acetate. A 1.2 mL sample of the eluted solution was analyzed by gas chromatography. Conversion was determined based on how much reactant had been consumed compared to the amount of reactant in the *t* = 0 chromatogram relative to the internal standard (biphenyl). Each reaction was run at least twice and the average conversions were reported. The error was calculated as either the difference from the actual value to the average (when comparing two runs) or one standard deviation (when comparing more than two runs).

##### Transfer Dehydrogenation of 4-Phenyl-2-butanol under a Trimethylamine Atmosphere

A solution of 4-phenyl-2-butanol (376 mg, 2.5 mmol, 1 equiv) and biphenyl (96 mg, 0.63 mmol, 0.25 equiv) was prepared in 5 mL acetone. A 25 μL aliquot was removed, diluted with 1 mL of acetone, and analyzed by gas chromatography to give the *t* = 0 chromatogram. Trimethylamine gas was bubbled through the solution for 60 s, and a balloon of trimethylamine was placed atop the condenser. Iron catalyst **7-MeO** (39 mg, 0.063 mmol, 2.5 mol %) was added to the reaction solution, which was placed in a 60 °C oil bath. Aliquots (200 μL) of the solution were removed at the desired times and diluted with 1 mL hexanes. Residual iron was removed from each aliquot by adding it to a Pasteur pipet half filled with silica gel and eluting with 4 mL 1:1 hexanes/ethyl acetate. A 1.2 mL sample of the eluted solution was analyzed by gas chromatography. Conversion was determined based on how much reactant had been consumed compared to the amount of reactant in the *t* = 0 chromatogram relative to the internal standard (biphenyl).

#### NMR Experiments

##### Transfer Dehydrogenation of 2-Heptanol

A solution of 2-heptanol (42 mg, 0.36 mmol), **6-Me** (21 mg, 0.038 mmol), anhydrous trimethylamine *N*-oxide (2.9 mg, 0.039 mmol), and 1,3,5-trimethoxybenzene (6.6 mg, 0.039 mmol) in acetone-*d*_6_ (0.75 mL) was stirred at rt in a round-bottom flask under nitrogen, turning from bright to dark red. After 10 min, the solution was transferred to a sealable, pressurizable (J Young) NMR tube under nitrogen. A ^1^H NMR spectrum (with a spectral range of 15 to −30 ppm) was taken at 298 K; the sample was heated in the spectrometer to 338 K, and ^1^H NMR spectra (with a spectral range of 15 to −30 ppm) were taken every 15 min over 45 min.

##### Transfer Dehydrogenation of 2-Heptanol Mimicking 75% Conversion

A solution of 2-heptanol (10 μL, 7.9 mg, 0.068 mmol, 0.25 equiv), 2-heptanone (28 μL, 23 mg, 0.20 mmol, 0.75 equiv), isopropanol (16 μL, 12 mg, 0.02 mmol, 0.75 equiv), **6-Me** (15 mg, 0.027 mmol, 0.1 equiv), anhydrous trimethylamine *N*-oxide (2.9 mg, 0.039 mmol, 0.14 equiv), and 1,3,5-trimethoxybenzene (4.6 mg, 0.027 mmol) in acetone-*d*_6_ (0.75 mL) was stirred at rt in a round-bottom flask under nitrogen, turning from bright to dark red. After 10 min, the solution was transferred to a sealable, pressurized (J Young) NMR tube under nitrogen. A ^1^H NMR spectrum (with a spectral range of 15 to −30 ppm) was taken at 300 K; the sample was heated in the spectrometer to 328 K, and ^1^H NMR spectra (with a spectral range of 15 to −30 ppm) were taken every 15 min over 60 min.

##### Transfer Hydrogenation of 2-Butanone

A solution of 2-butanone (19.6 mg, 0.27 mmol), **6-Me** (15 mg, 0.027 mmol), anhydrous trimethylamine *N*-oxide (4.1 mg, 0.054 mmol), and 1,3,5-trimethoxybenzene (4.6 mg, 0.027 mmol) in 1:1 isopropanol/benzene-*d*_6_ (0.7 mL total) was stirred at rt in a round-bottom flask under nitrogen, turning from red to dark brown. After 10 min, the solution was transferred to a sealable, pressurizable (J Young) NMR tube under nitrogen. A ^1^H NMR spectrum (with a spectral range of 15 to −30 ppm) was taken at 298 K; the sample was heated in the spectrometer to 338 K, and ^1^H NMR spectra (with a spectral range of 15 to −30 ppm) were taken every 15 min over 45 min.

##### Transfer Hydrogenation of 2-Butanone Mimicking 75% Conversion

A solution of 2-butanone (6.0 μL, 4.9 mg, 0.068 mmol, 0.25 equiv), 2-butanol (19 μL, 15 mg, 0.20 mmol, 0.75 equiv), acetone (15 μL, 12 mg, 0.20 mmol, 0.75 equiv), **6-Me** (15 mg, 0.027 mmol, 0.1 equiv), anhydrous trimethylamine *N*-oxide (2.7 mg, 0.035 mmol, 0.13 equiv), and 1,3,5-trimethoxybenzene (4.6 mg, 0.027 mmol) in 1:1 isopropanol/benzene-*d*_6_ (0.8 mL total) was stirred at rt in a round-bottom flask under nitrogen, turning from red to dark brown. After 5 min, the solution was transferred to a sealable, pressurized (J Young) NMR tube under nitrogen. A ^1^H NMR spectrum (with a spectral range of 15 to −30 ppm) was taken at 300 K; the sample was heated in the spectrometer to 338 K, and ^1^H NMR spectra (with a spectral range of 15 to −30 ppm) were taken every 15 min over 30 min.

## Supporting Information Available

Progress plots for reactions run under an atmosphere of trimethylamine, data processing details for KIE and Hammett studies, and NMR spectra (PDF)
