# Supplementary material for: Substituent Effects and Mechanistic Insights on the Catalytic Activities of (Tetraarylcyclopentadienone)iron Carbonyl Compounds in Transfer Hydrogenations and Dehydrogenations
Source: Organometallics. 2023 Oct 5;42(21):3053–65. doi: 10.1021/acs.organomet.3c00284 (PMC10647929; doi:10.1021/acs.organomet.3c00284)

Supporting Information

Substituent Effects and Mechanistic Insights on the Catalytic Activities of (Tetraarylcyclopentadienone)iron Carbonyl Compounds in Transfer Hydrogenations and Dehydrogenations

Bryn K. Werley,† Xintong Hou,† Evan P. Bertonazzi,† Anthony Chianese,‡ and Timothy W. Funk*†

†Department of Chemistry, Gettysburg College, Gettysburg, Pennsylvania, 17325, United States

‡Department of Chemistry, Colgate University, Hamilton, New York, 13346, United States

Contents:

**Transfer dehydrogenation and hydrogenation under a trimethylamine atmosphere (Figures S1 and S2)**	S2
**Hammett plot data processing**	S3
**Kinetic isotope effect data processing in acetophenone transfer hydrogenations**	S3
**NMR spectra (Figures S3–S28)**	S4–S29

Figure S1. Transfer dehydrogenation of 4-phenyl-2-butanol with and without excess trimethylamine

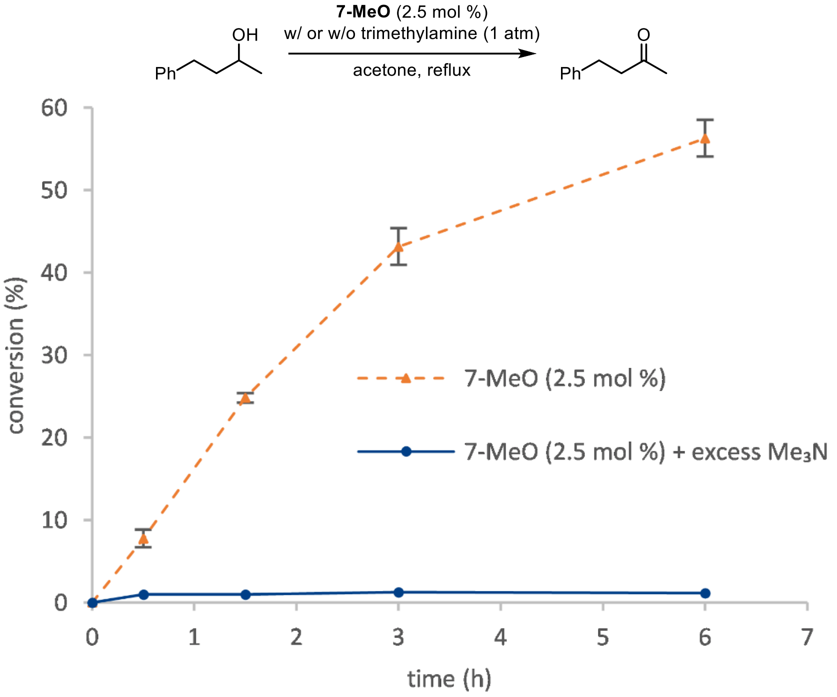

Conversion (%) vs. time (h) for the transfer dehydrogenation of 4-phenyl-2-butanol under typical reaction conditions (dashed orange line) or under an atmosphere of trimethylamine (solid blue line) using **7-MeO** (2.5 mol %). Conversions were determined by GC relative to biphenyl.

Figure S2. Transfer hydrogenation of acetophenone with and without excess trimethylamine

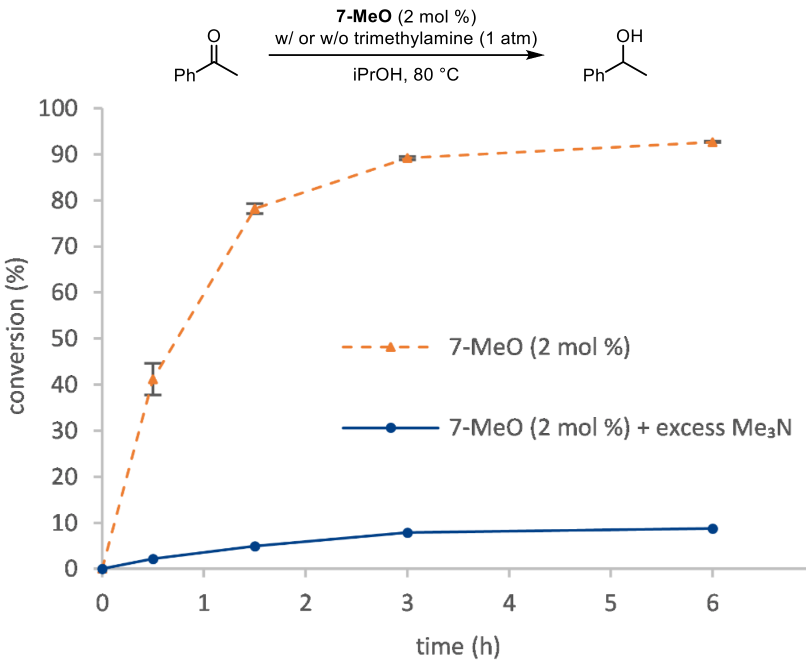

Conversion (%) vs. time (h) for the transfer hydrogenation of acetophenone under typical reaction conditions (dashed orange line) or under an atmosphere of trimethylamine (solid blue line) using **7-MeO** (2 mol %). Conversions were determined by GC relative to biphenyl.

Hammett plot data processing

The transfer hydrogenation and dehydrogenation reactions were run as described in the Experimental section. Plots of conversion vs. time were made using the first few points (linear region at low conversions) for each individual run, and the data was fit with a linear trendline using Microsoft Excel. The slope of the line was used as the initial rate, and the average of the initial rates for each set of replicates was calculated. The average initial rate for the unsubstituted catalyst or substrate (e.g., **6-H**, acetophenone, 1-phenylethanol) was defined as k_H, and the rates of substituted catalysts or substrates were called k. Errors in the average initial rates were calculated as either the difference from the individual initial rates to the average (when comparing two runs) or one standard deviation (when comparing more than two runs). These errors were propagated through the data processing.

Kinetic isotope effect data processing in acetophenone transfer hydrogenations

Transfer hydrogenation reactions of acetophenone in isopropanol and isopropanol-d_8 were run as described in the "Transfer hydrogenations monitored over <24 h" in the Experimental section. Plots of conversion vs. time were made using the first few points (linear region at low conversions) for each individual run (Figure S1), and the linear parts of the data were fit with a linear trendline using Microsoft Excel (Figure S2). The slope of the line was used as the initial rate, and the average of the initial rates for each pair of replicates was calculated. The average initial rate for the reaction run in isopropanol was defined as k_H, and the average initial rate for the reaction run in isopropanol-d_8 was defined as k_D. Errors in the average initial rates were calculated as the difference from the individual initial rates to the average. These errors were propagated through the data processing.

NMR Spectra

All ^{1}H and ^{13}C{^{1}H} NMR spectra were recorded at ambient temperature at 400 MHz and 100 MHz, respectively, on a Bruker Avance Neo 400 MHz FT-NMR spectrometer unless otherwise noted. Chemical shifts are reported in parts per million (ppm) relative to tetramethylsilane (TMS) for spectra taken in $CDCl_3$. ^{1}H NMR spectra taken in acetone-d_6 and benzene-d_6 used the residual solvent peaks, 2.05 ppm and 7.16 ppm, respectively, as references. Samples were prepared in 0.7 mL of solvent unless otherwise noted.

Figure S3. ^{1}H NMR spectrum (400 MHz, $CDCl_3$) of **4-Cl**

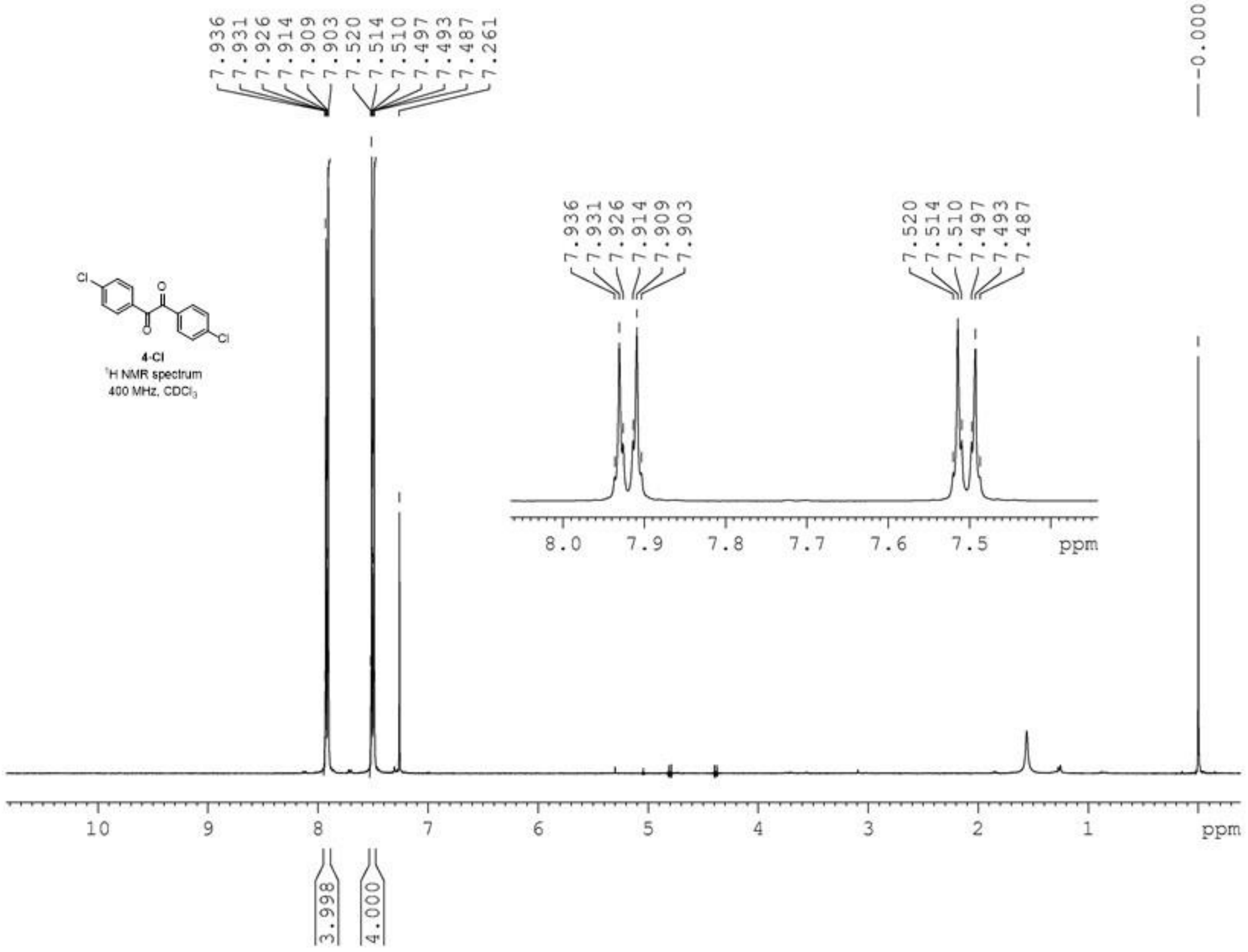

Figure S4. $^{13}C\{^{1}H\}$ NMR spectrum (100 MHz, $CDCl_3$) of **4-Cl**

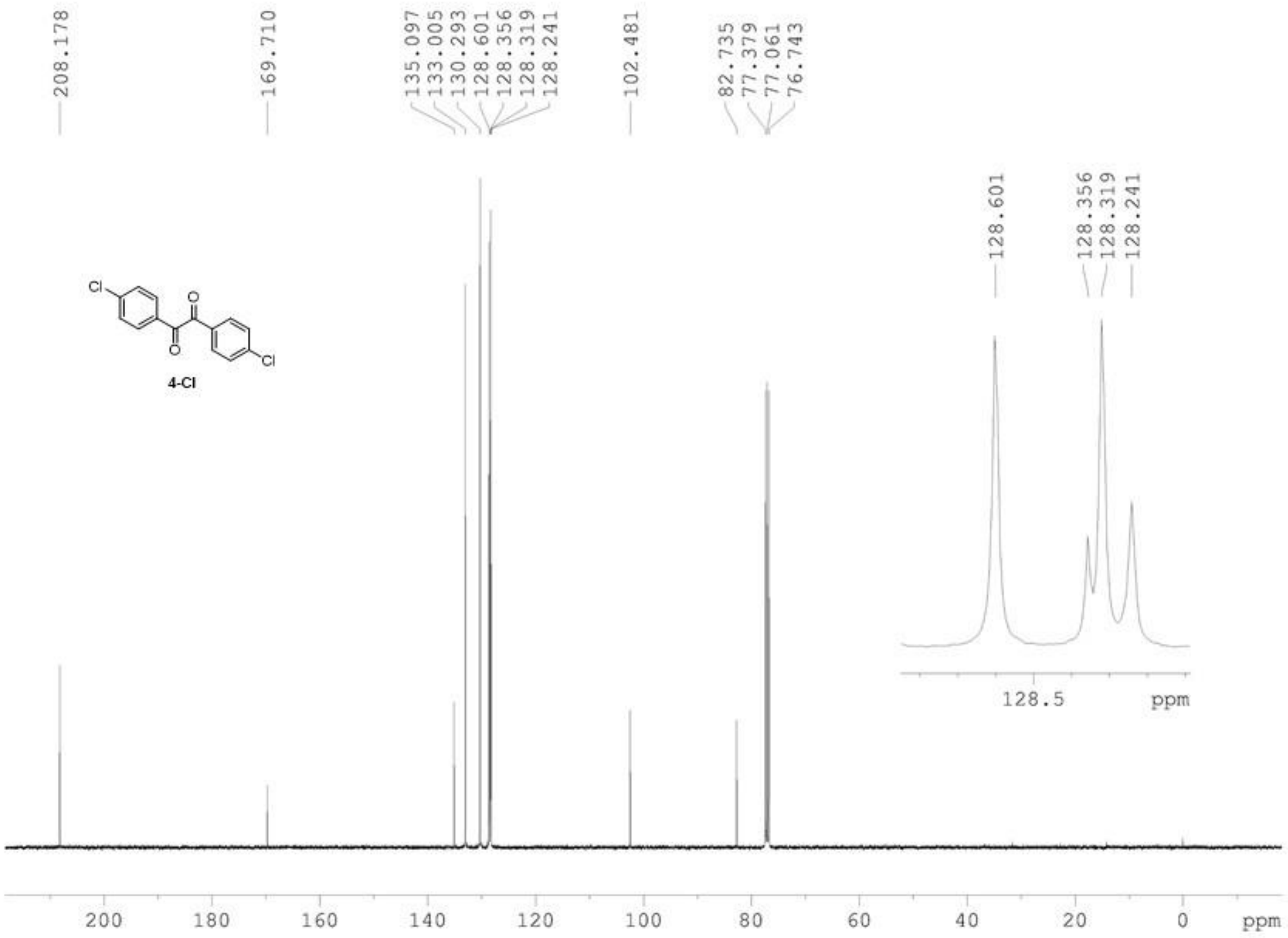

Figure S5. ^{1}H NMR spectrum (400 MHz, $CDCl_3$) of **4-CF₃**

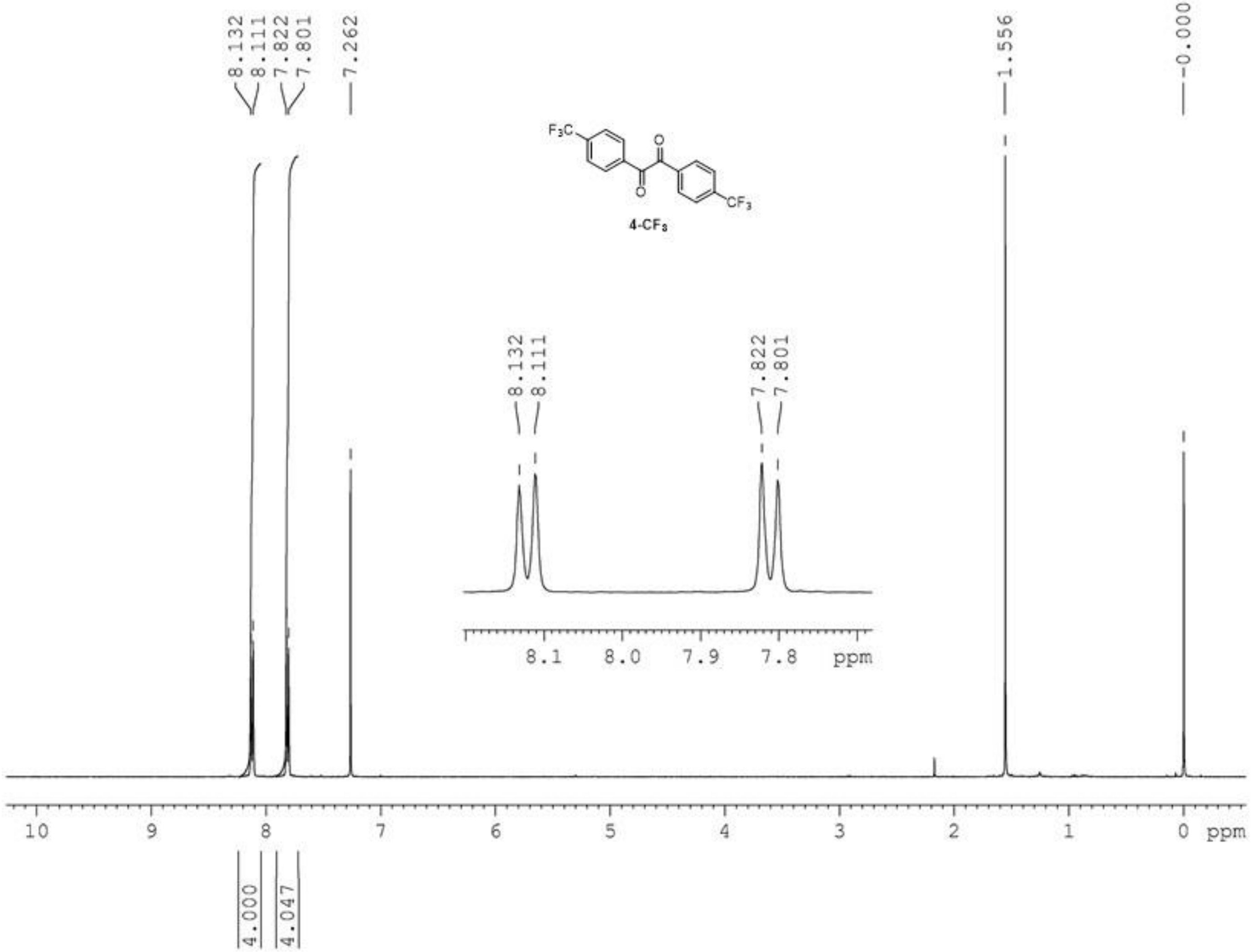

Figure S6. $^{13}C\{^{1}H\}$ NMR spectrum (100 MHz, $CDCl_3$) of **4-CF₃**

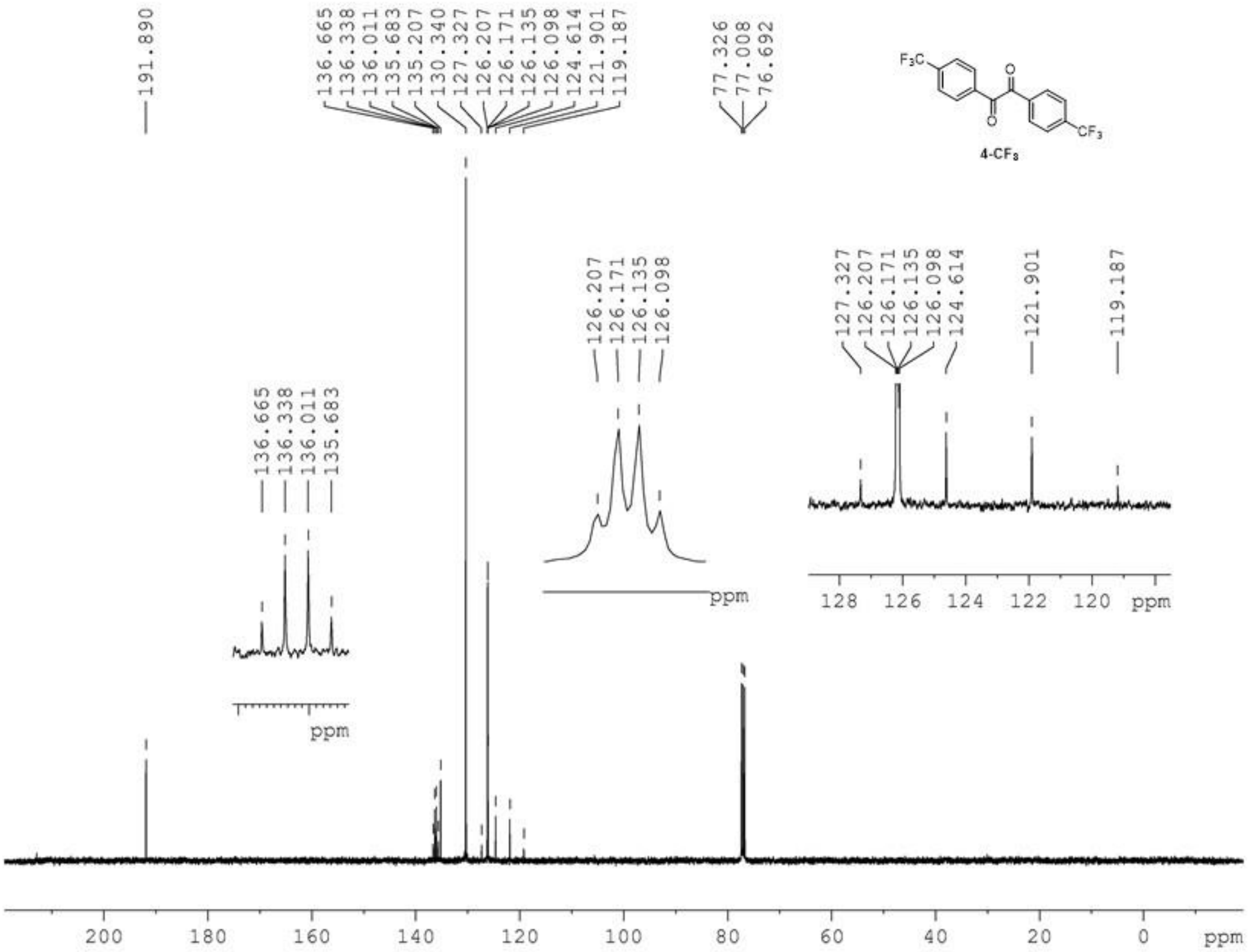

Figure S7. ^{1}H NMR spectrum (400 MHz, $CDCl_3$) of **5-Cl**

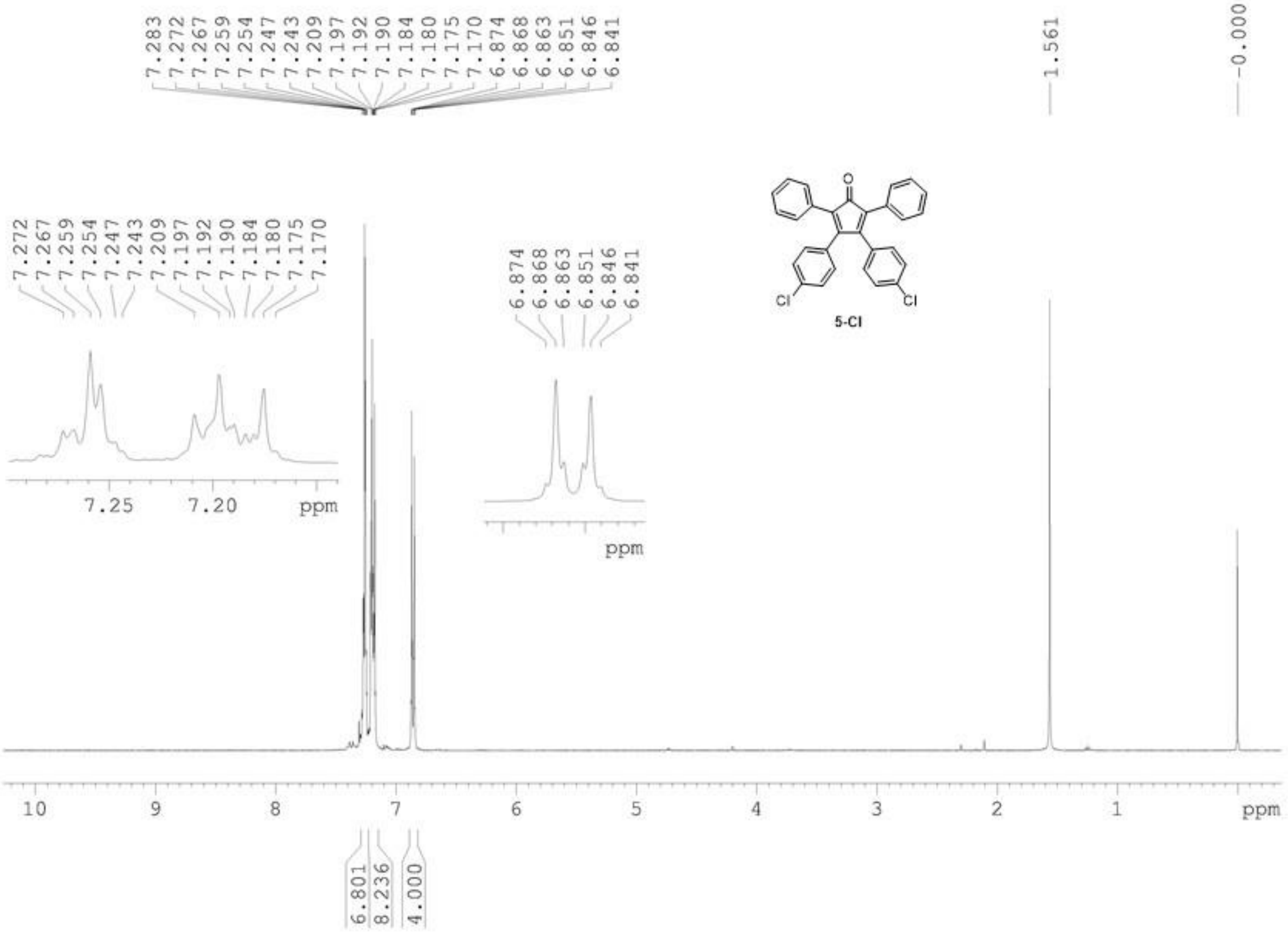

Figure S8. $^{13}C\{^1H\}$ NMR spectrum (100 MHz, $CDCl_3$) of **5-Cl**

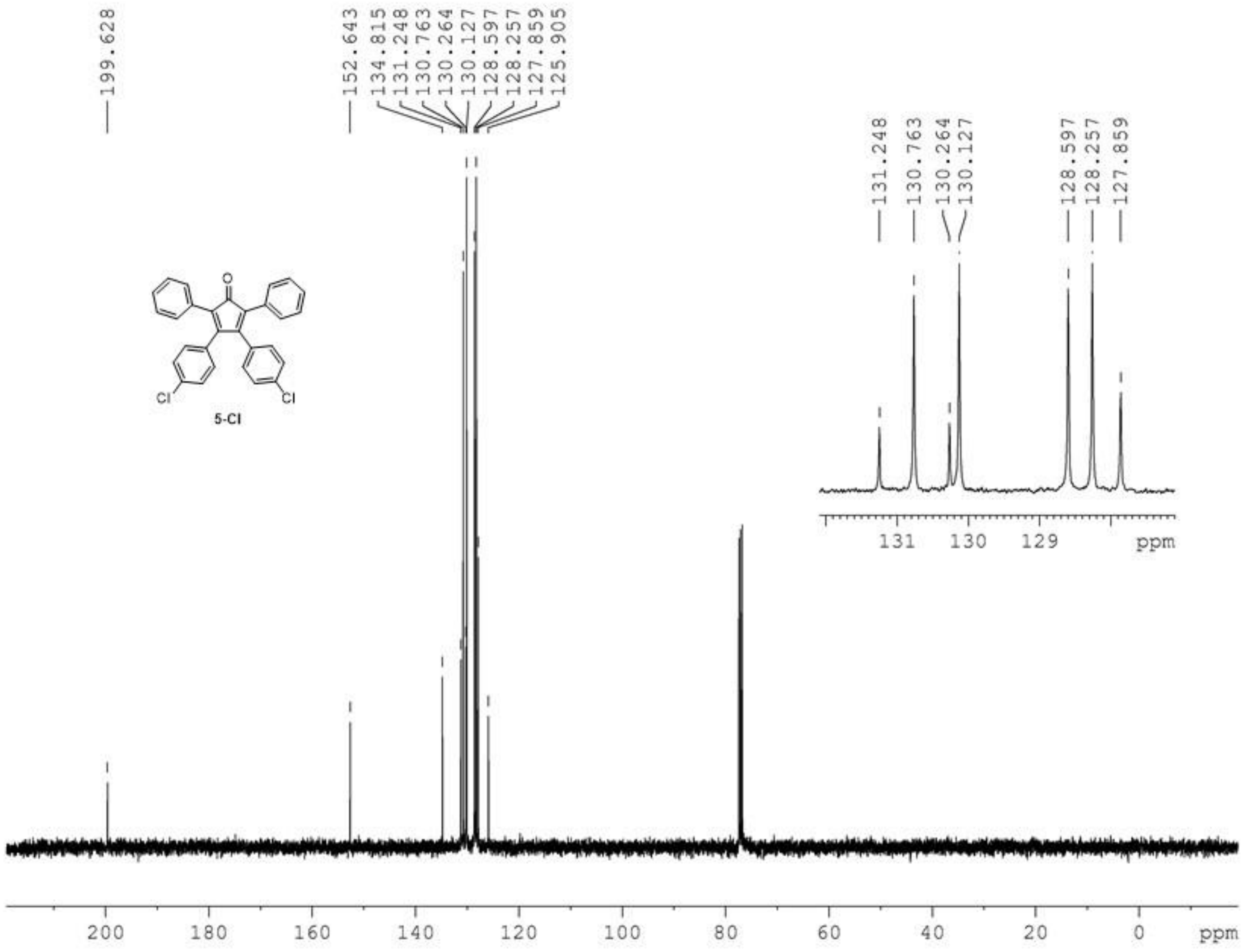

Figure S9. ^{1}H NMR spectrum (400 MHz, $CDCl_3$) of **5-CF₃**

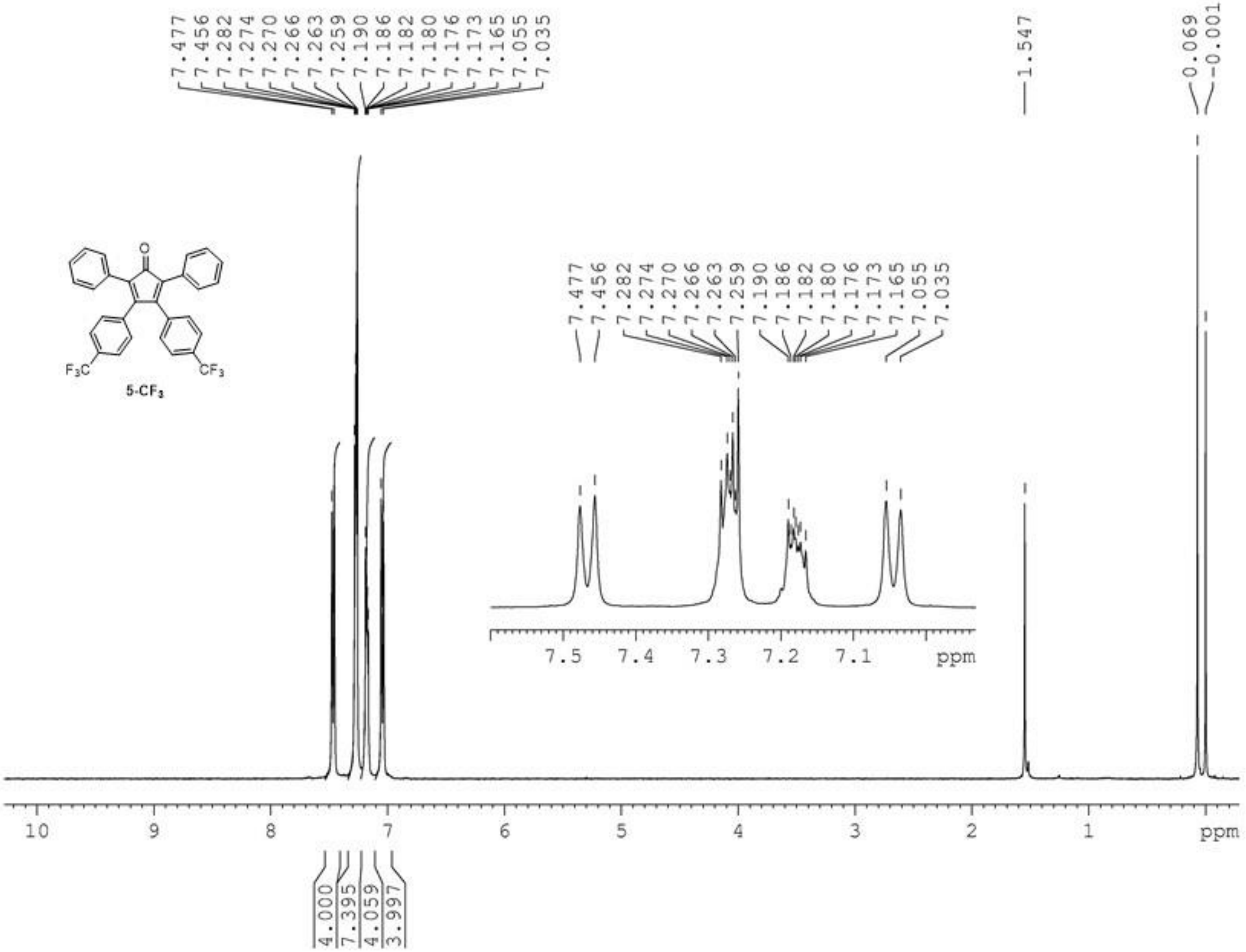

Figure S10. $^{13}C\{^{1}H\}$ NMR spectrum (100 MHz, $CDCl_3$) of **5-CF$_3$**

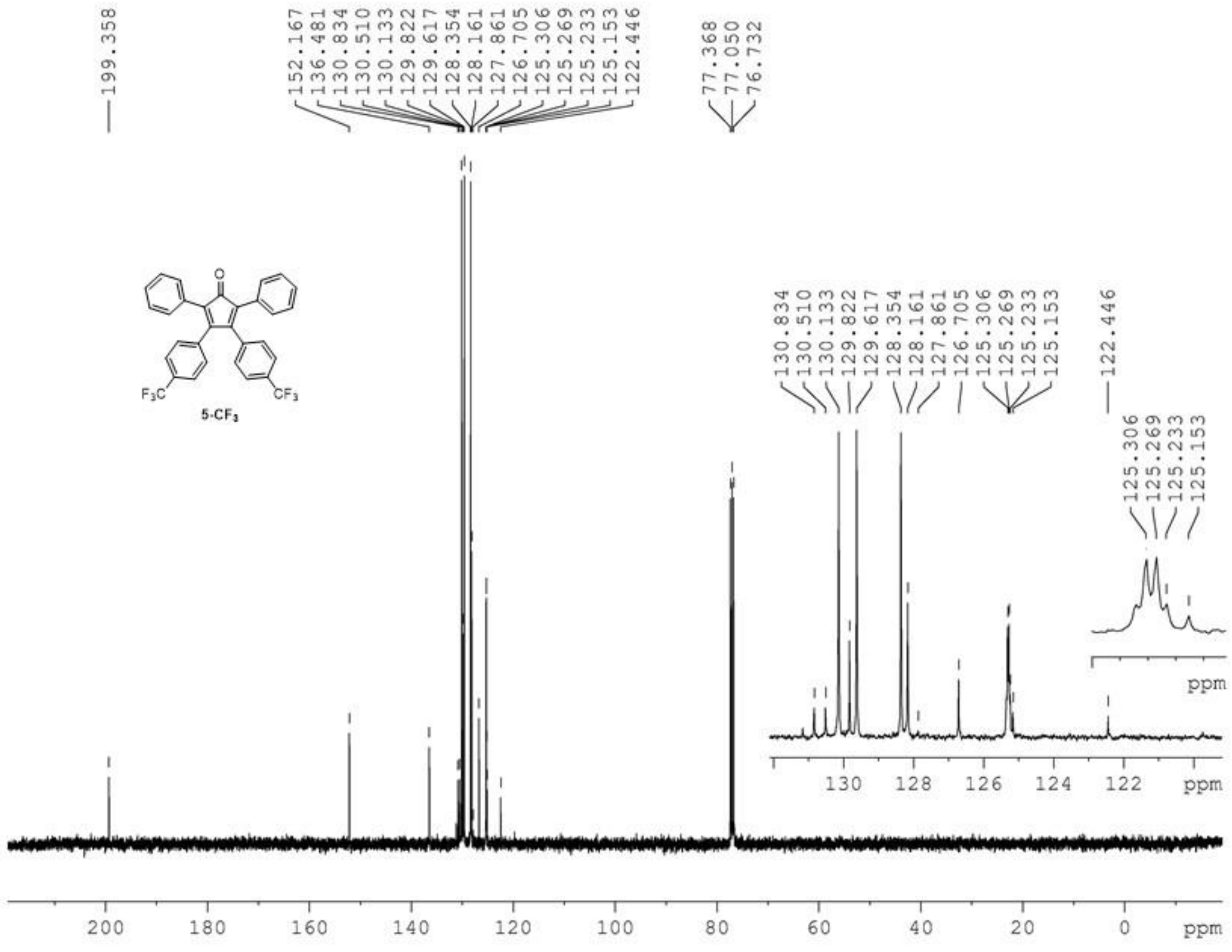

Figure S11. ^1H NMR spectrum (400 MHz, $CDCl_3$) of **6-Cl**

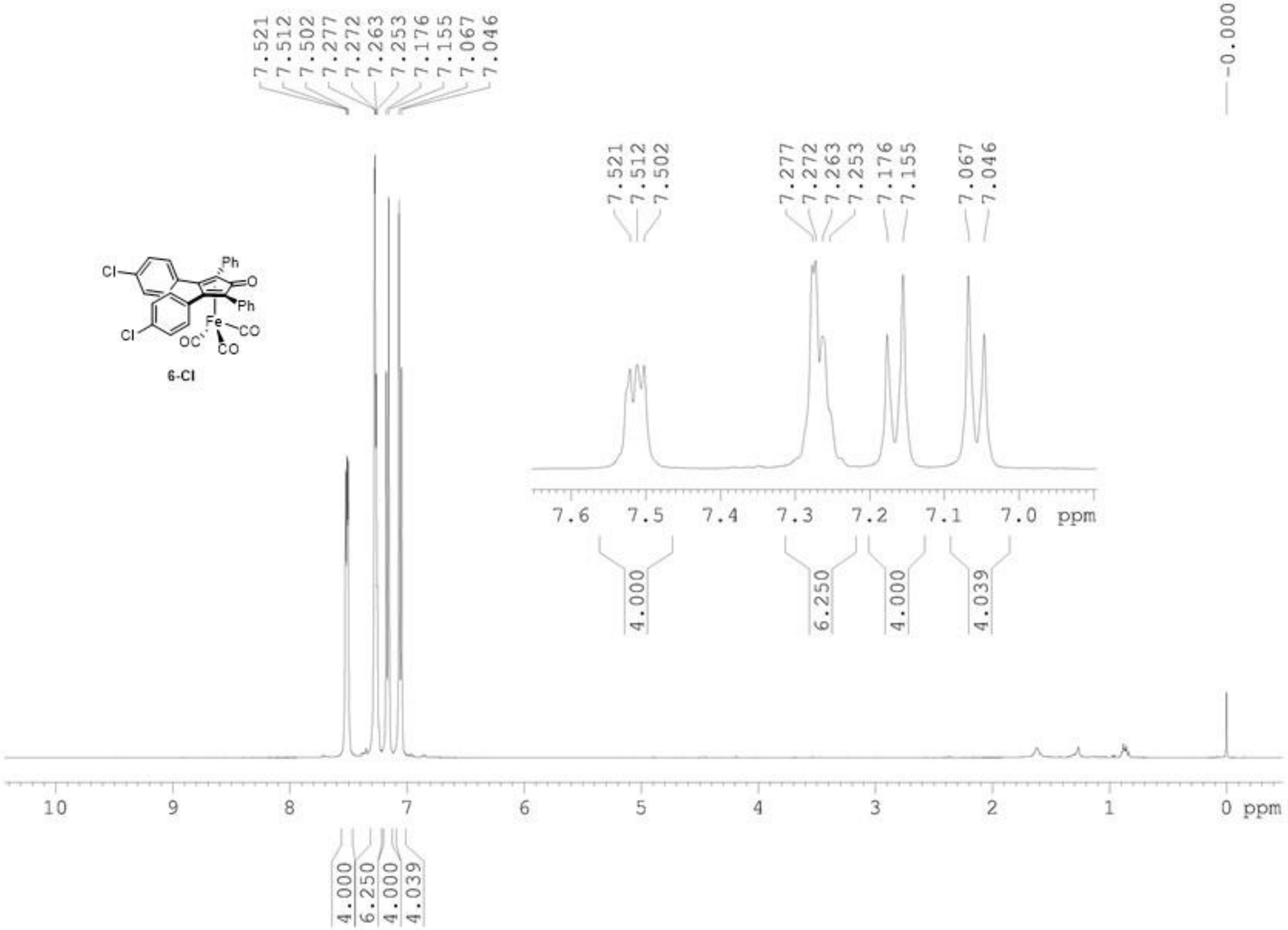

Figure S12. $^{13}C\{^{1}H\}$ NMR spectrum (100 MHz, $CDCl_3$) of **6-Cl**

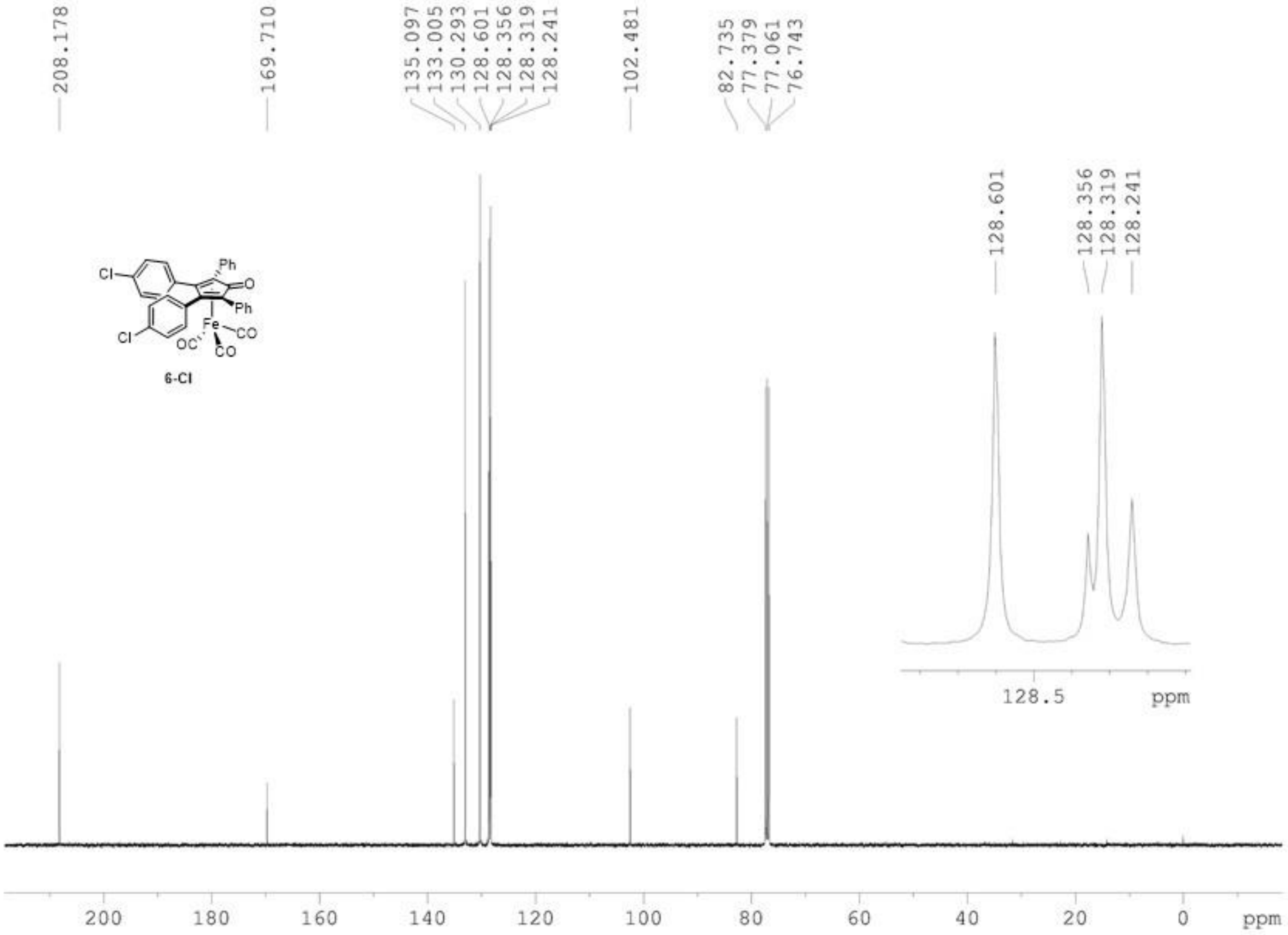

Figure S13. ^{1}H NMR spectrum (400 MHz, $CDCl_3$) of **6-CF$_3$**

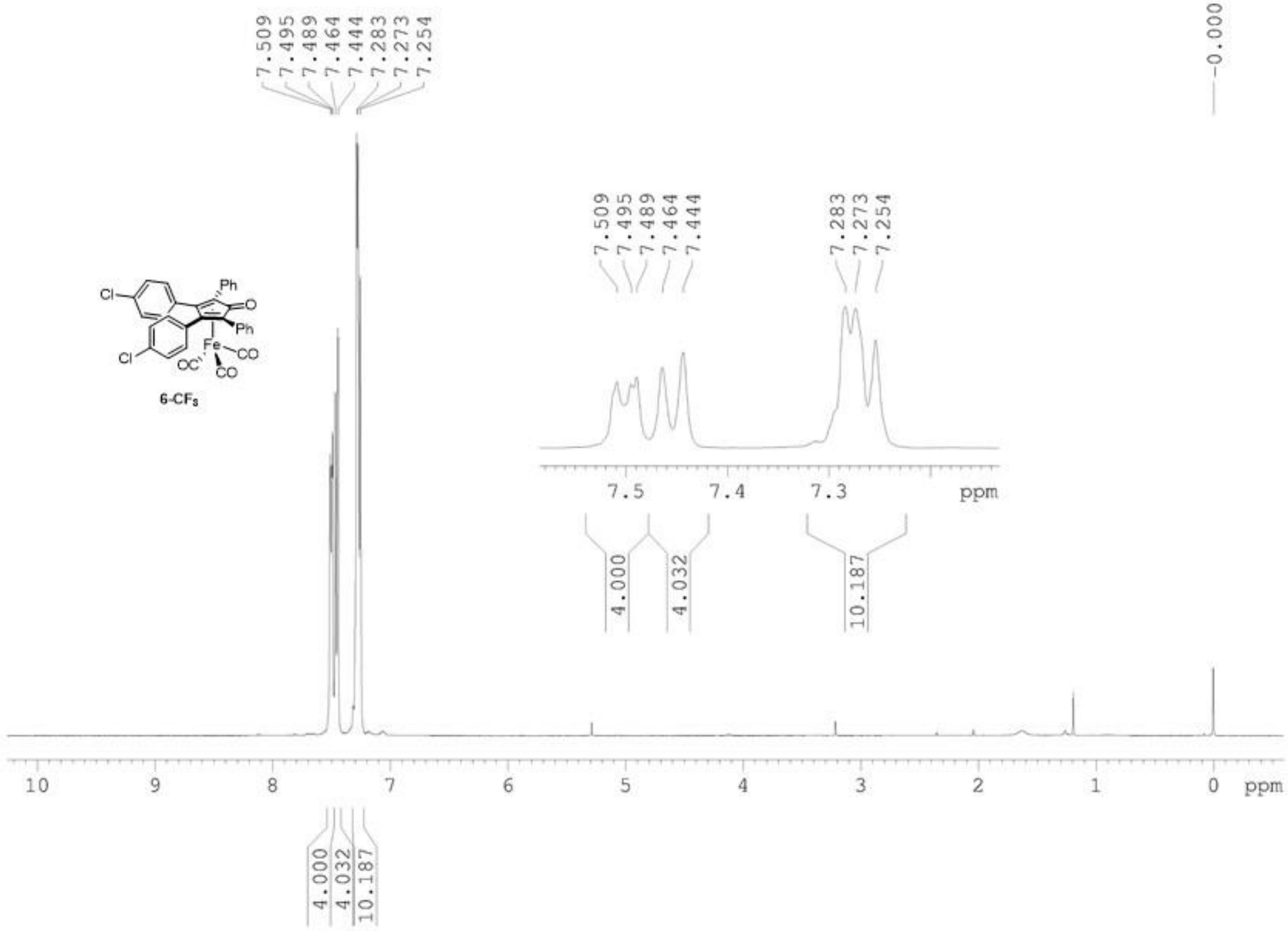

6-CF$_3$

Figure S14 $^{13}C\{^{1}H\}$ NMR spectrum (100 MHz, $CDCl_3$) of **6-CF$_3$**

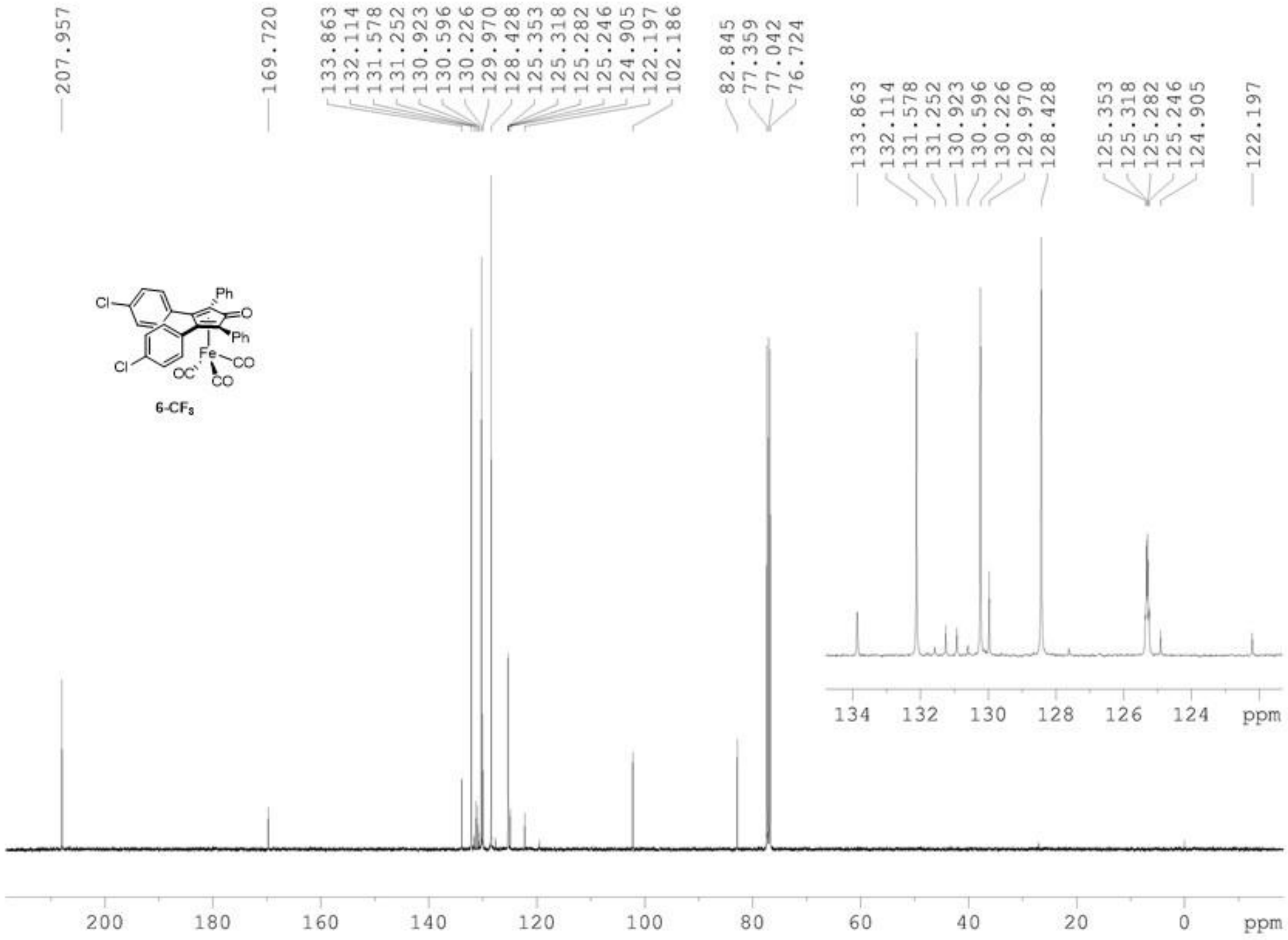

Figure S15. ^{1}H NMR spectrum (400 MHz, $CDCl_3$) of **7-MeO**

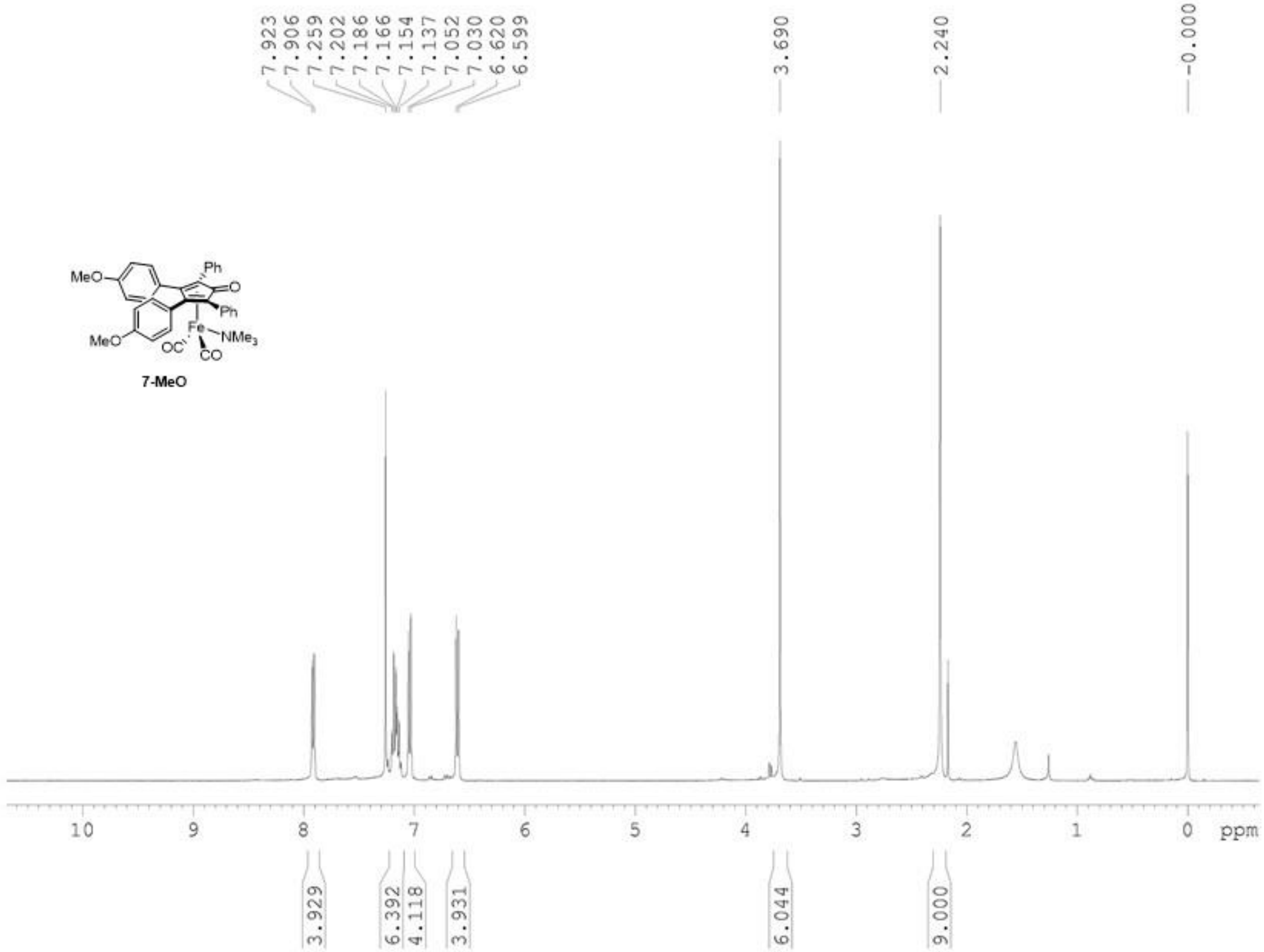

Figure S16. $^{13}C\{^{1}H\}$ NMR spectrum (100 MHz, $CDCl_3$) of **7-MeO**

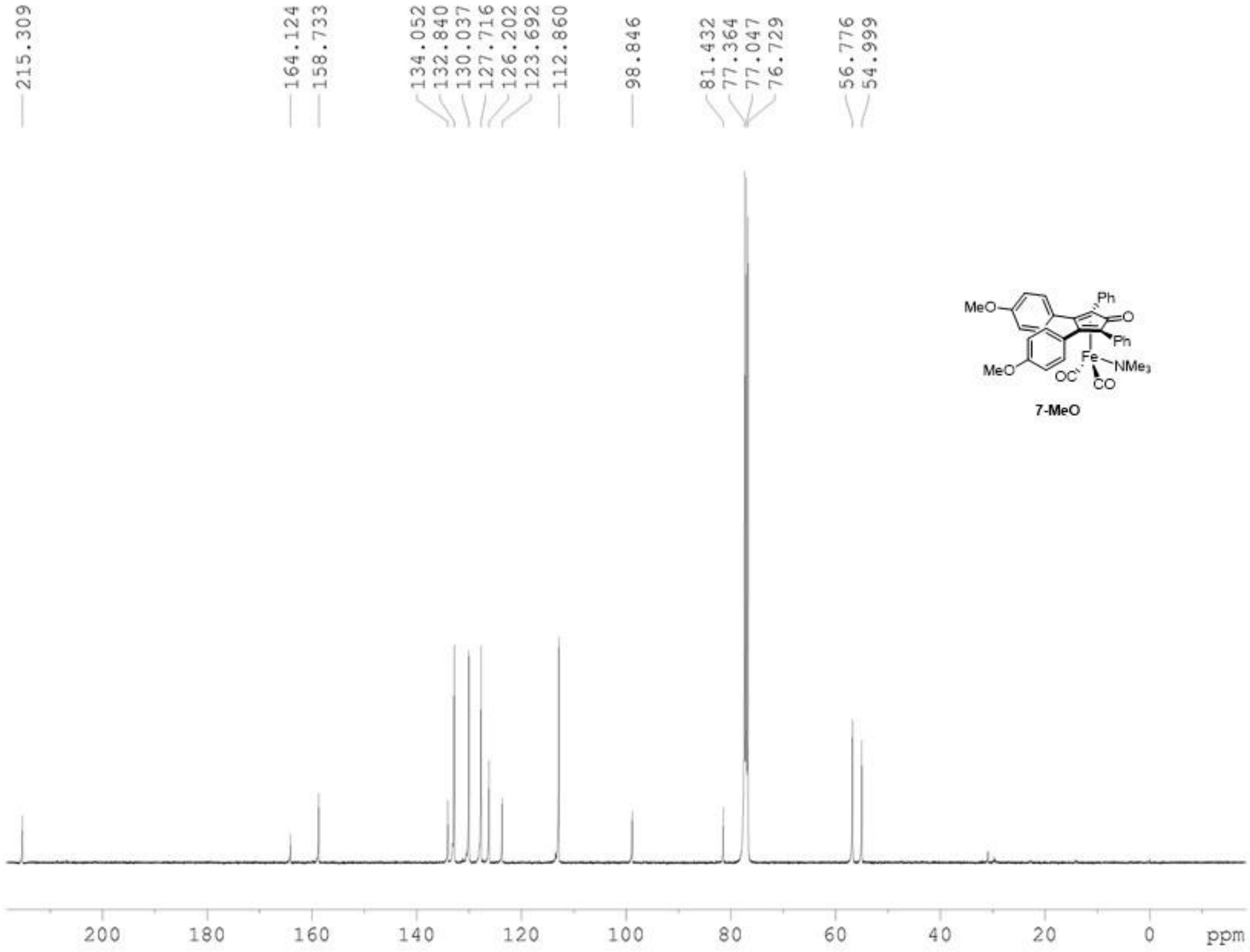

Figure S17. ^{1}H NMR spectrum (400 MHz, acetone-d_6) of **6-Me**

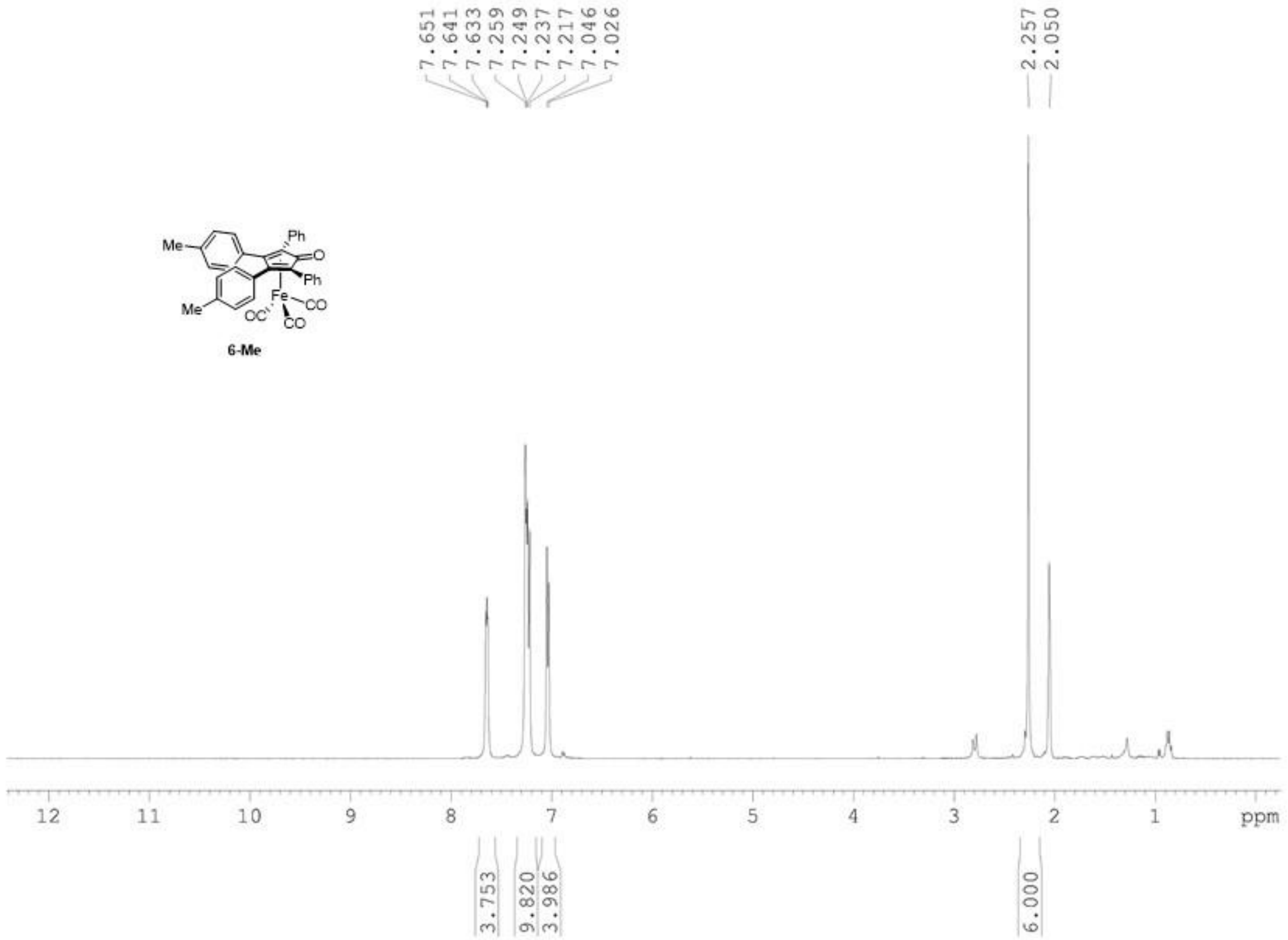

Figure S18. ^{1}H NMR spectrum (400 MHz, acetone-d_6) of **6-Me** (14.7 mg, 0.027 mmol) + anhydrous Me_3NO (2.4 mg, 0.032 mmol)

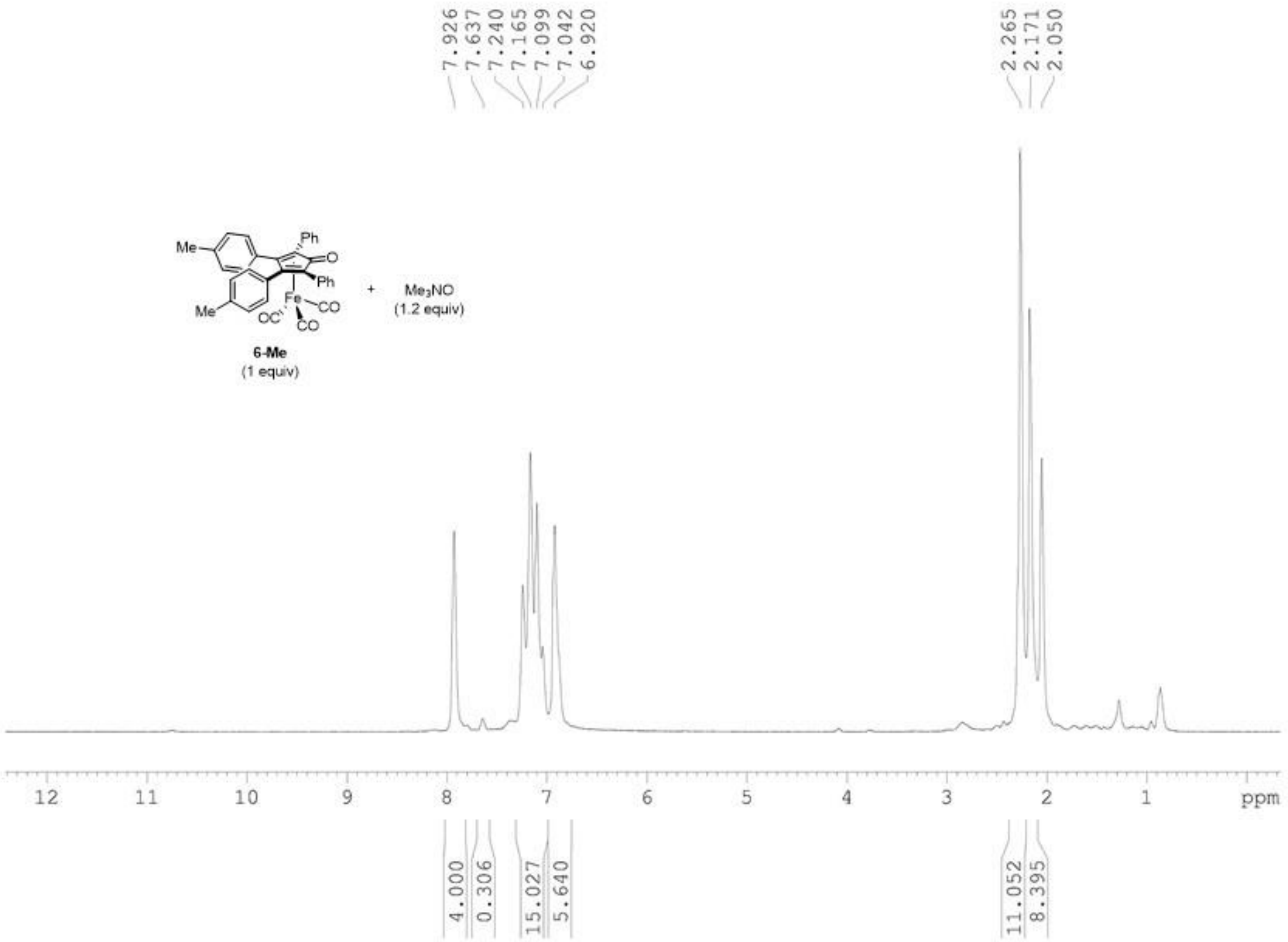

Figure S19. ^{1}H NMR spectrum (400 MHz, acetone-d_6) of transfer dehydrogenation of 2-heptanol with **6-Me** + Me_3NO. Spectrum was taken at rt after 10 minutes at rt. No peaks were observed up to 15 ppm or down to –30 ppm.

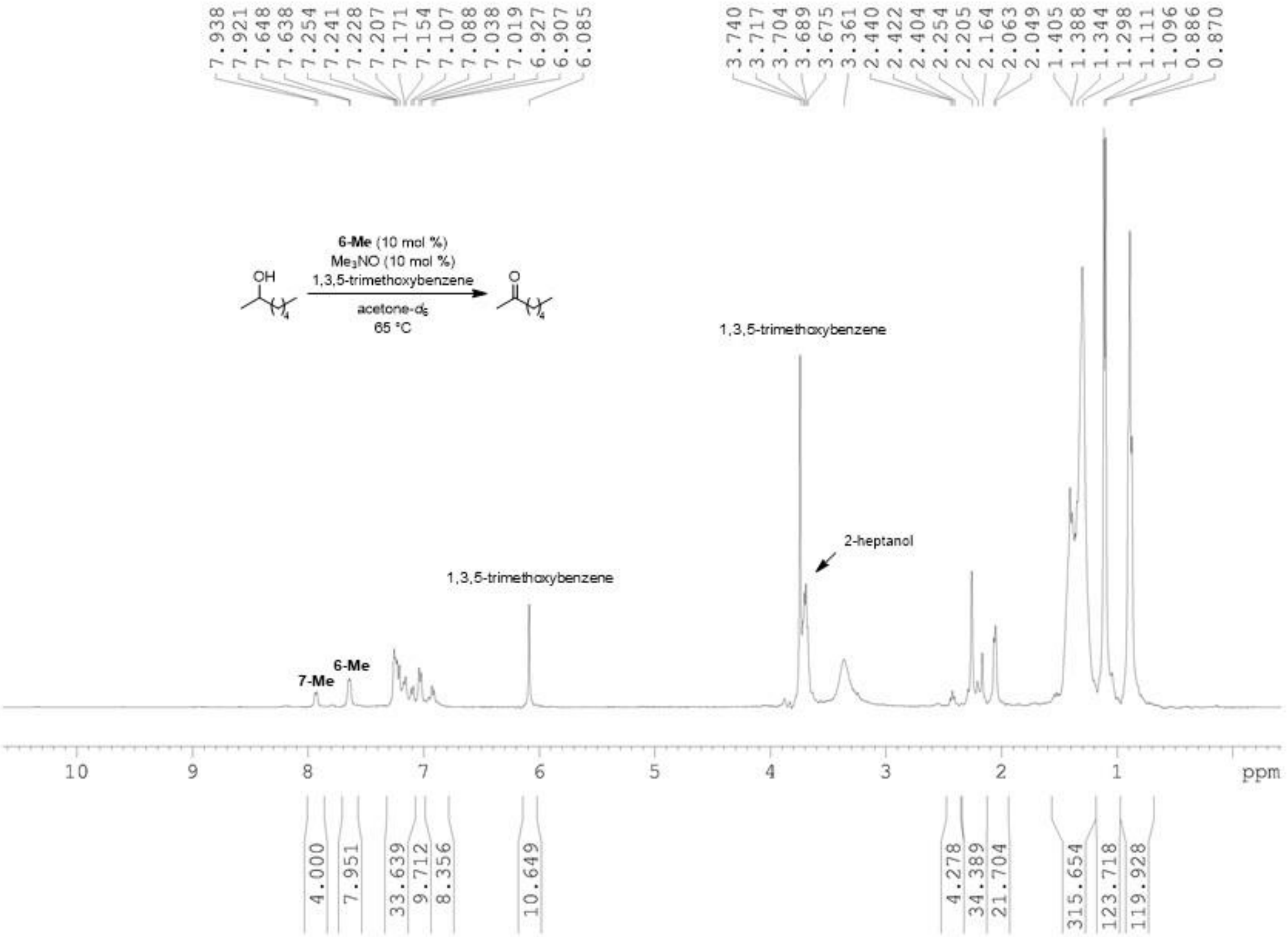

Figure S20. ^{1}H NMR spectrum (400 MHz, acetone-d_6) of transfer dehydrogenation of 2-heptanol with **6-Me** + Me_3NO. Spectrum was taken at 65 °C after 45 minutes at 65 °C. No peaks were observed up to 15 ppm or down to –30 ppm.

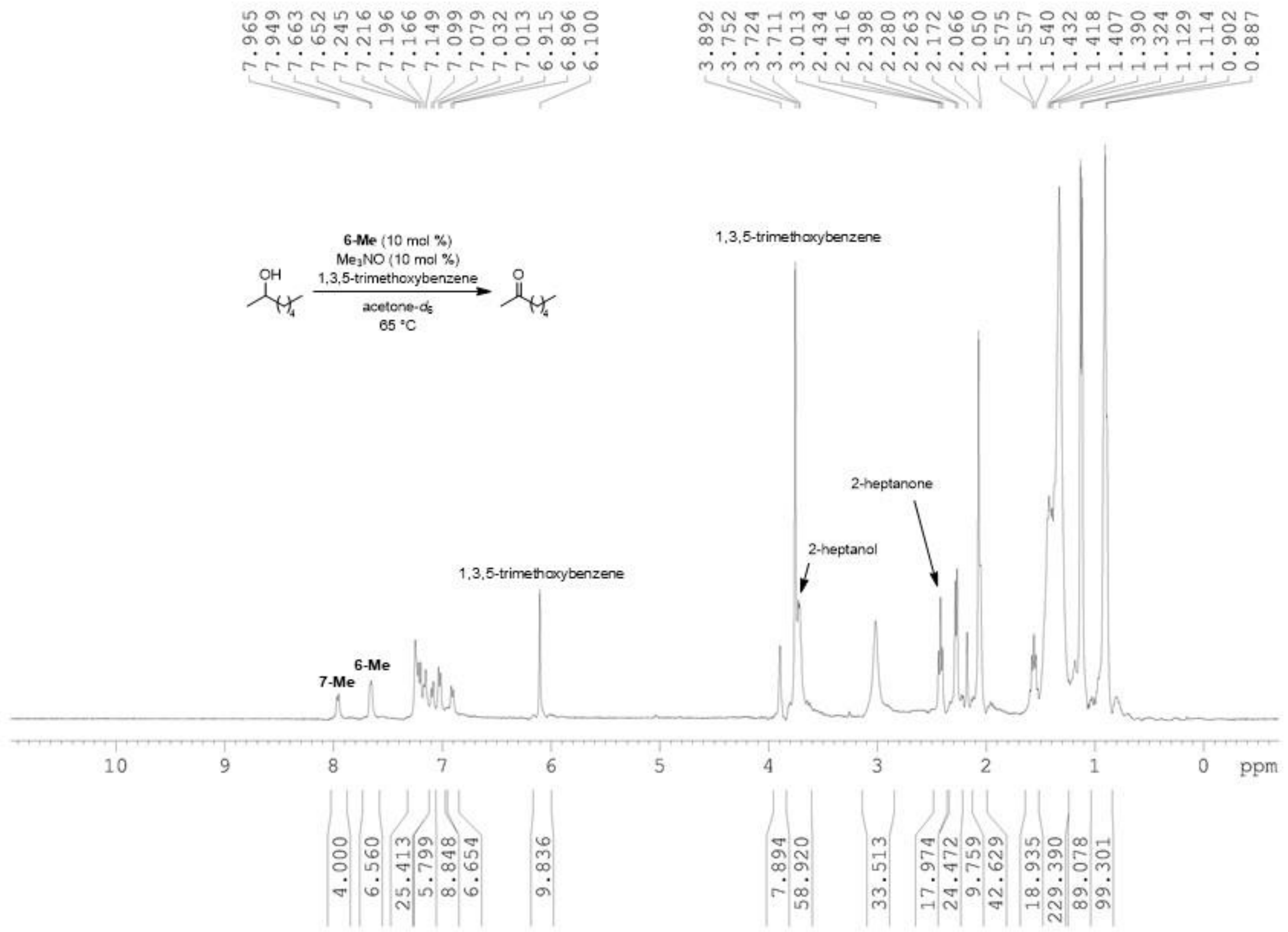

Figure S21. ^{1}H NMR spectrum (400 MHz, acetone-d_6) of transfer dehydrogenation of 2-heptanol with **6-Me** + Me_3NO mimicking the conditions at 75% conversion (see Experimental Section for details). Spectrum was taken at rt after 10 minutes at rt. No peaks were observed up to 15 ppm or down to –30 ppm other than the small peak at approximately –11 ppm.

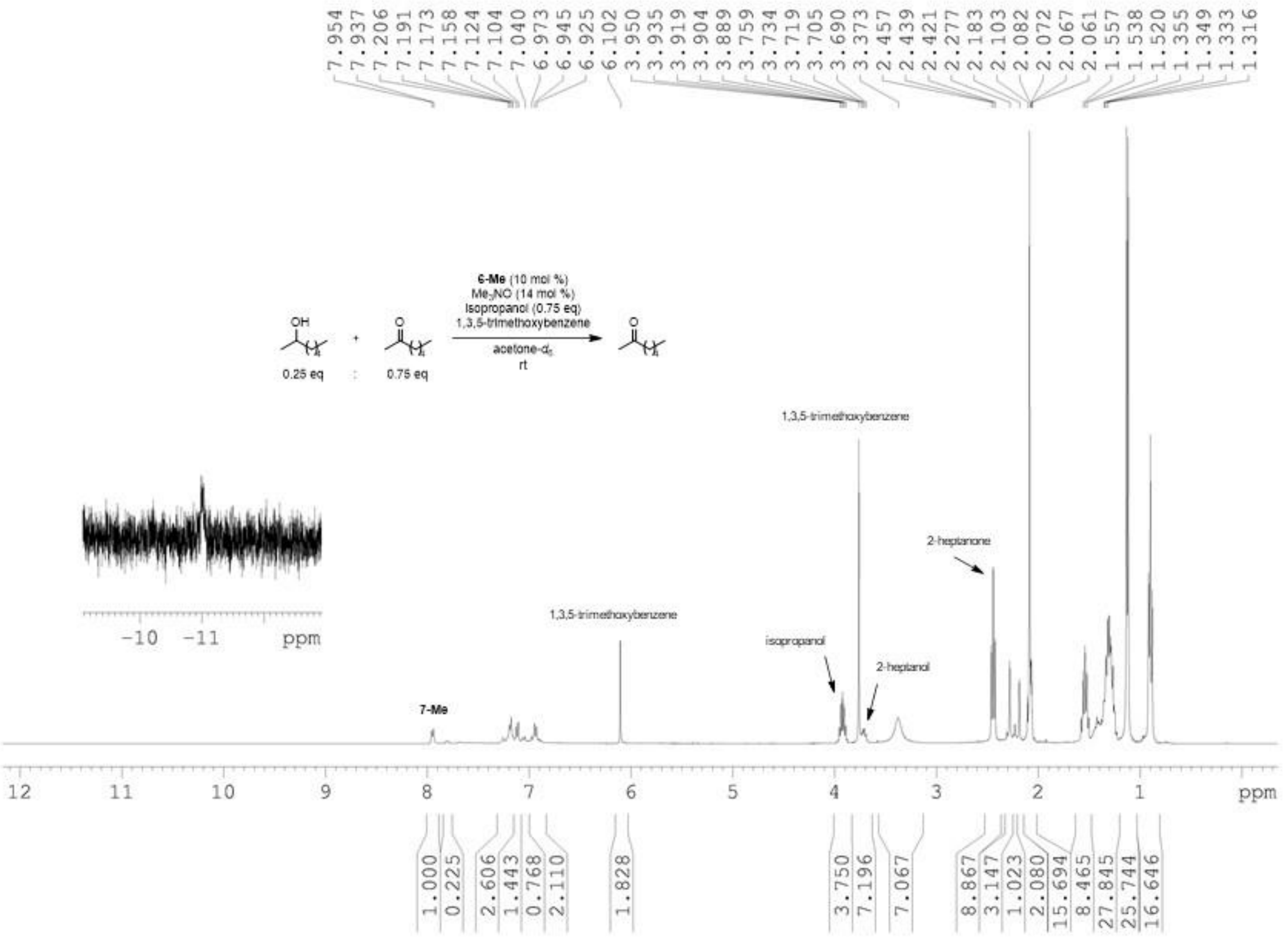

Figure S22. ^{1}H NMR spectrum (400 MHz, acetone-d_6) of transfer dehydrogenation of 2-heptanol with **6-Me** + Me_3NO mimicking the conditions at 75% conversion (see Experimental Section for details). Spectrum was taken at 55 °C after 45 minutes at 55 °C. No peaks were observed up to 15 ppm or down to –30 ppm.

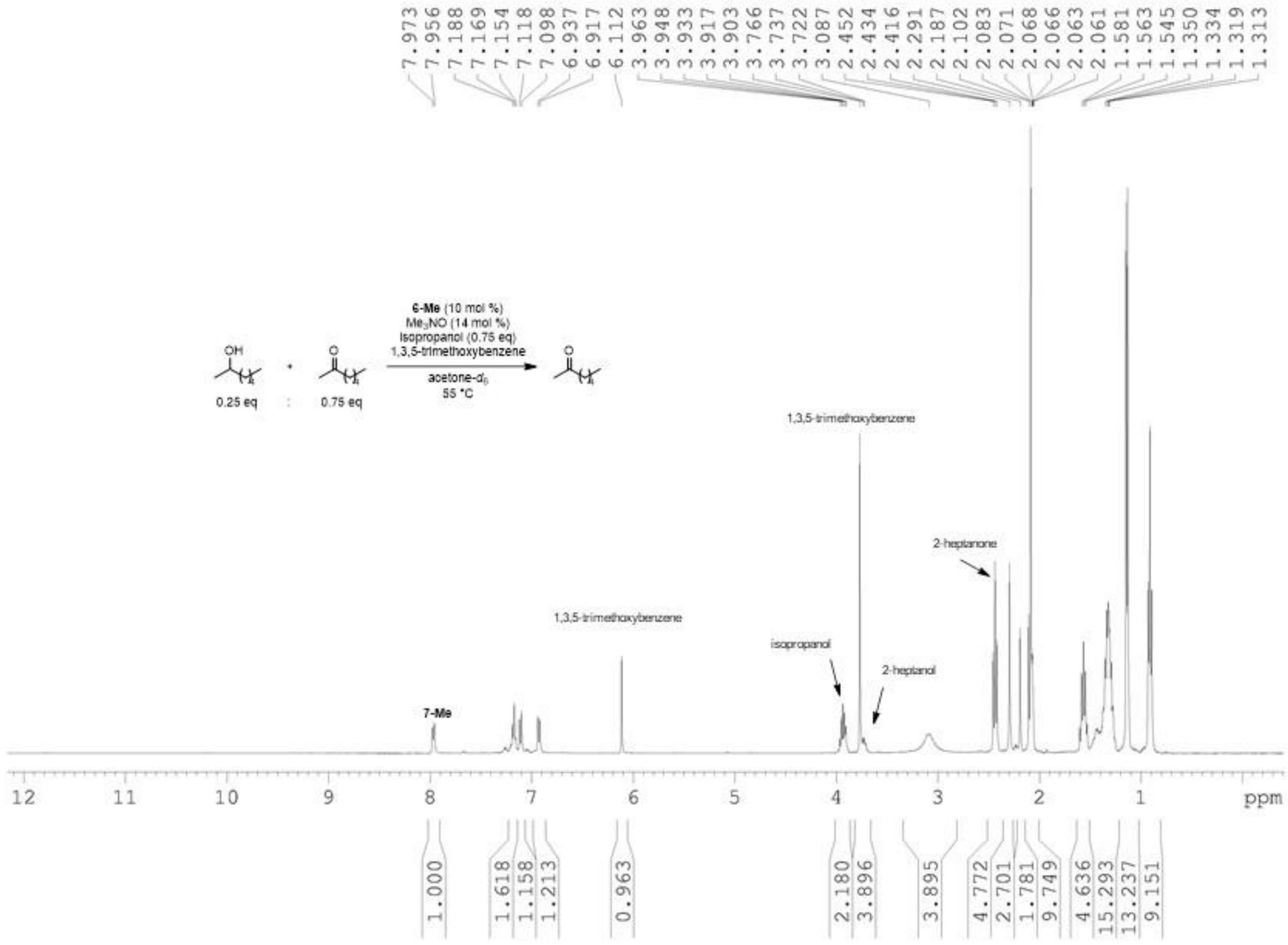

Figure S23. ^{1}H NMR spectrum (400 MHz, 1:1 benzene-d_6 and isopropanol) of **6-Me**.

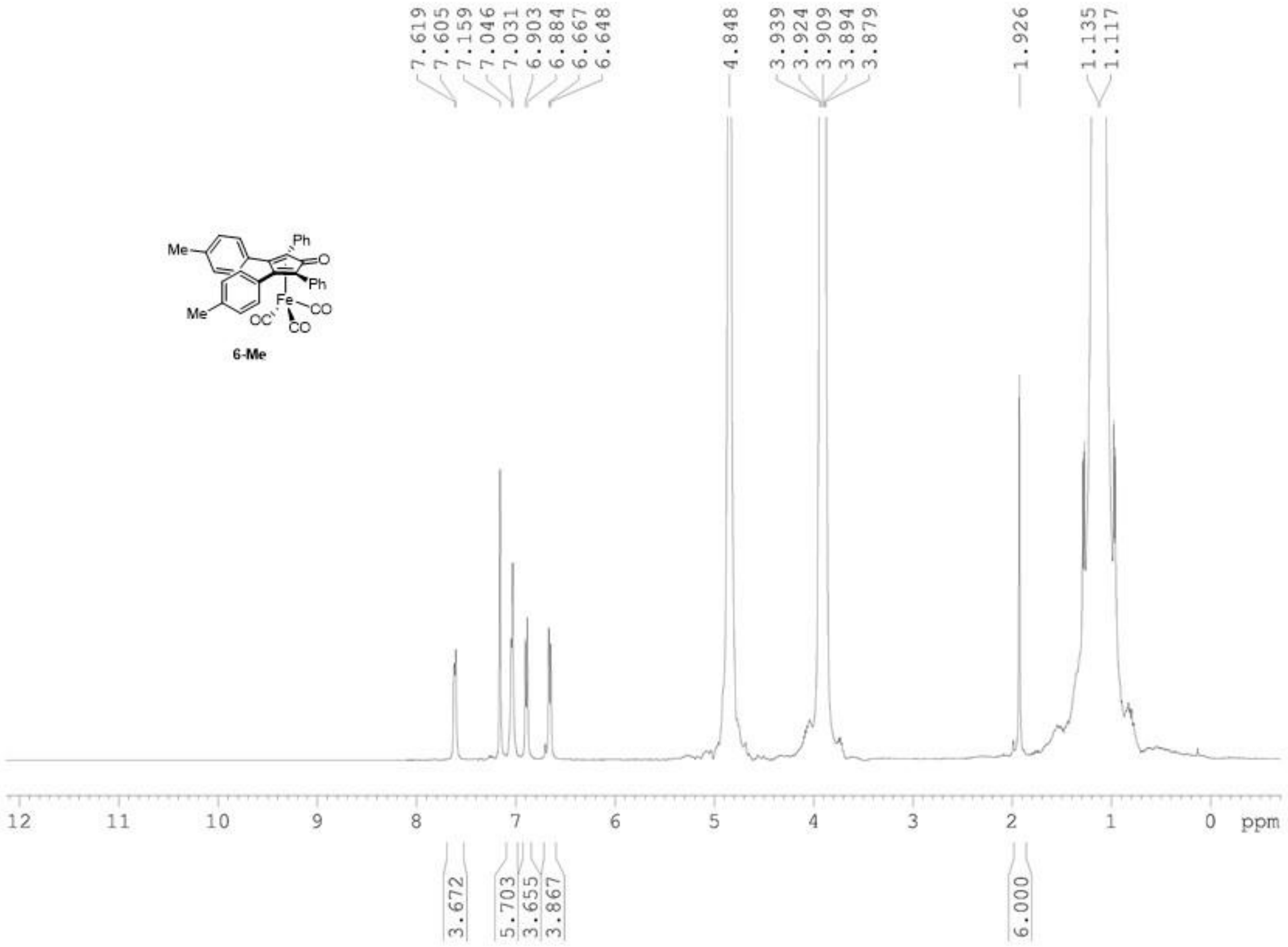

Figure S24. ^{1}H NMR spectrum (400 MHz, 1:1 benzene-d_6 and isopropanol) of **6-Me** (14.7 mg, 0.027 mmol) + anhydrous Me_3NO (2.0 mg, 0.027 mmol) at rt. No peaks other than the singlet at –10.72 ppm were observed down to –30 ppm.

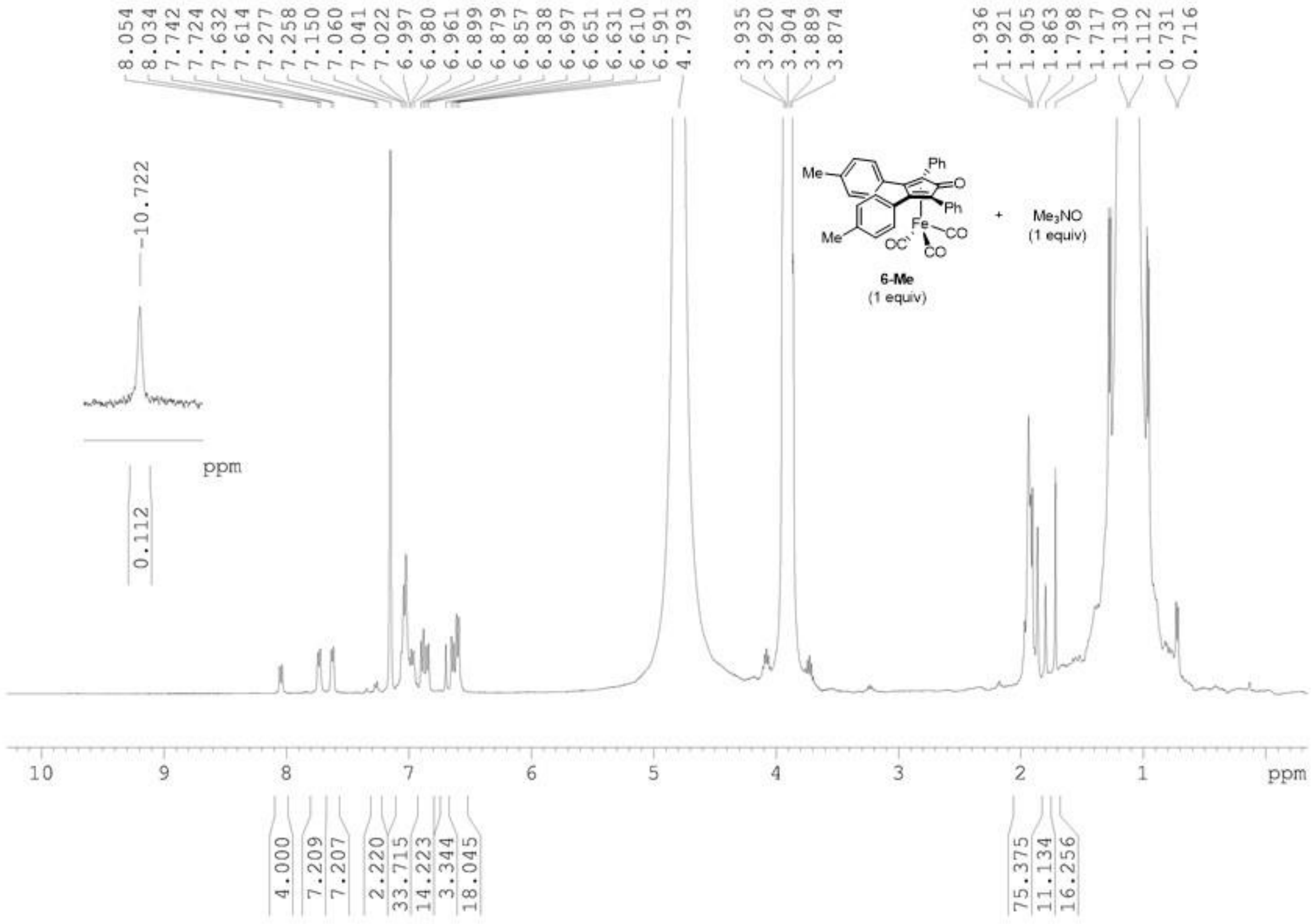

Figure S25. ^{1}H NMR spectrum (400 MHz, 1:1 benzene-d_6 and isopropanol) of **6-Me** (14.7 mg, 0.027 mmol) + anhydrous Me_3NO (2.0 mg, 0.027 mmol). Spectrum was taken at 65 °C after 10 minutes at 65 °C. No peaks other than the singlet at –10.72 ppm were observed down to –30 ppm.

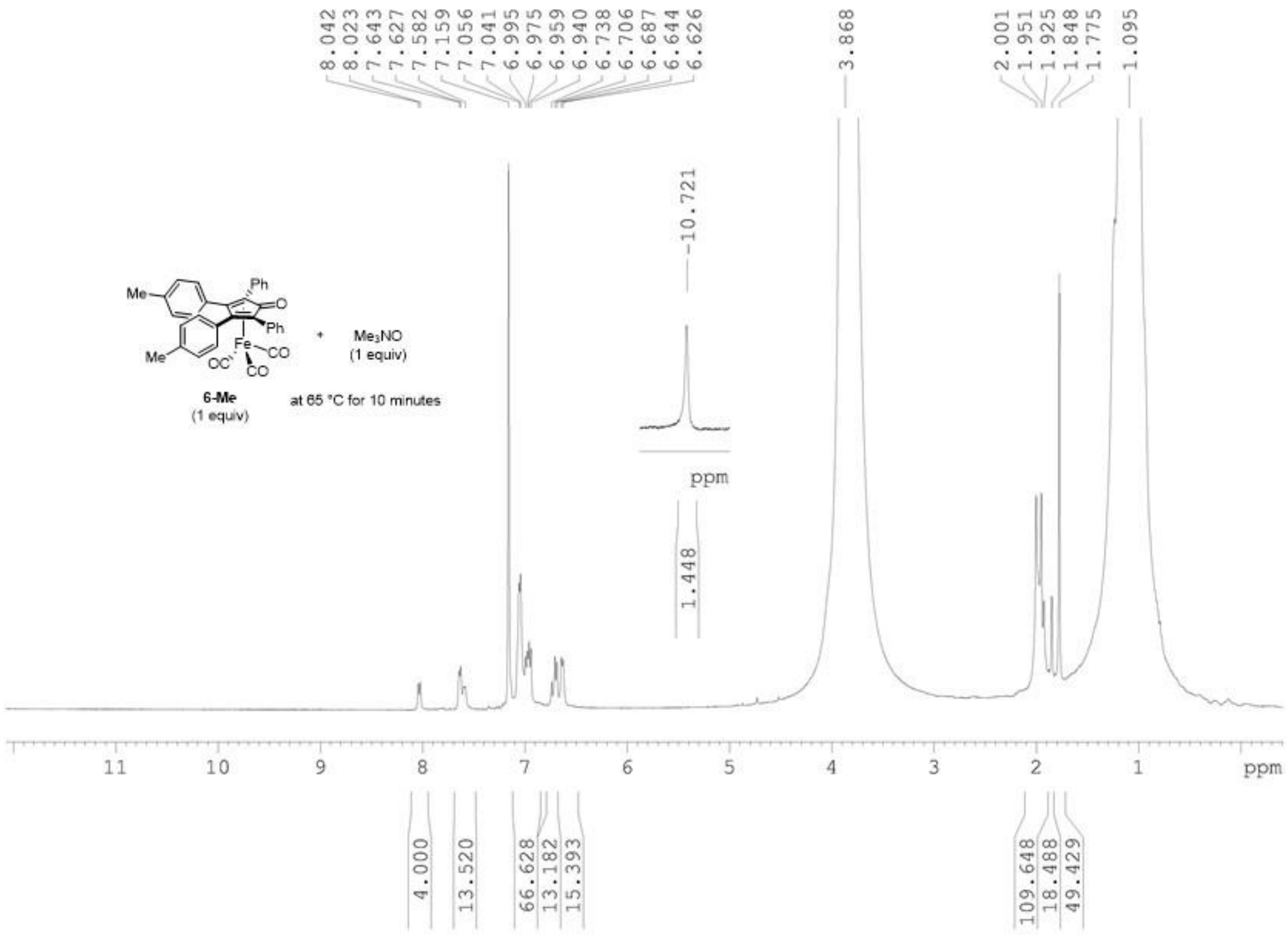

Figure S26. ^{1}H NMR spectrum (400 MHz, 1:1 benzene-d_6 and isopropanol) of transfer hydrogenation of 2-butanone with 1:2 **6-Me** + Me_3NO. Spectrum was taken at 65 °C after 5 minutes at 65 °C. No peaks were observed up to 15 ppm or down to –30 ppm other than those shown.

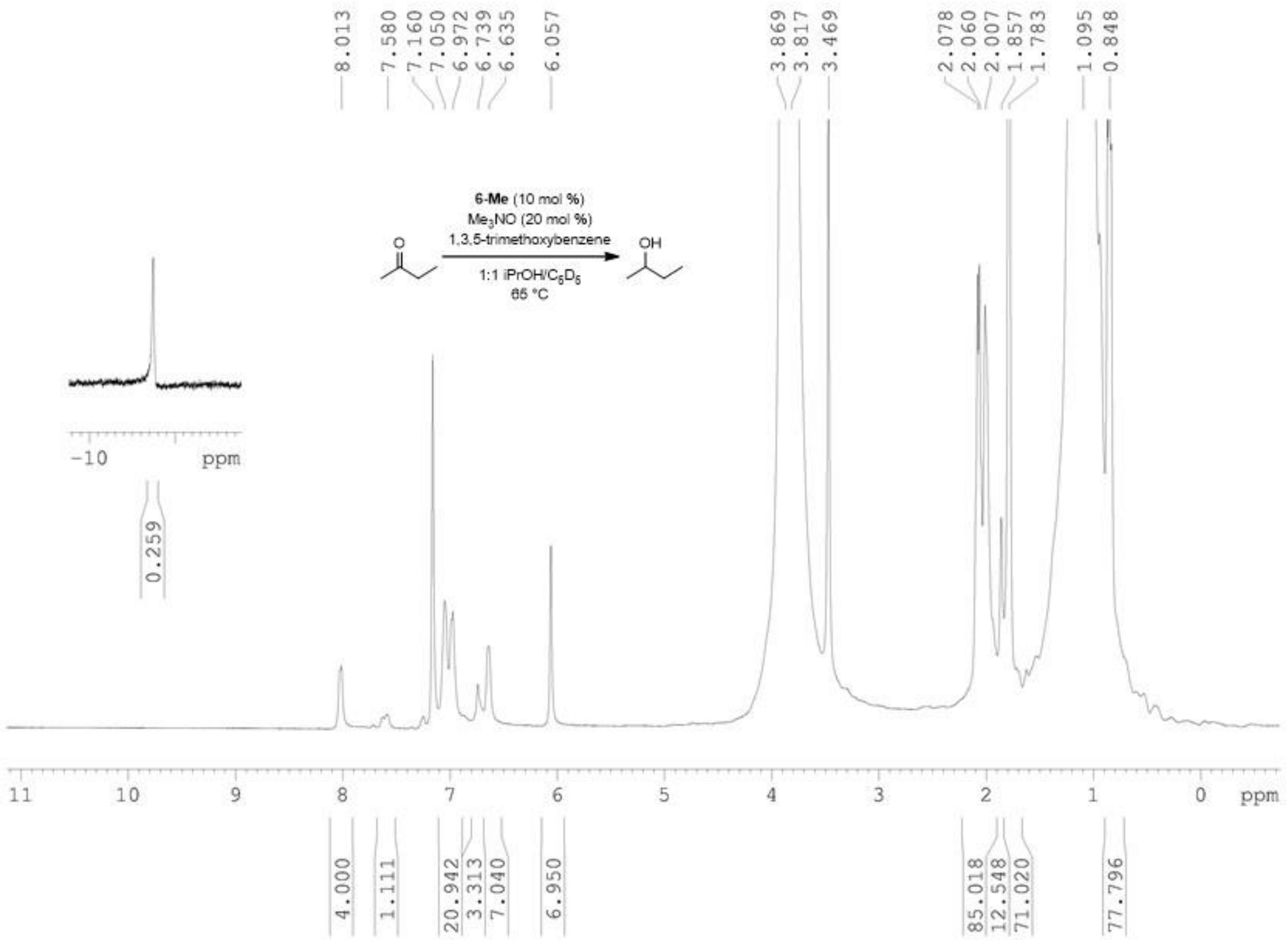

Figure S27. ^{1}H NMR spectrum (400 MHz, 1:1 benzene-d_6 and isopropanol) of transfer hydrogenation of 2-butanone with **6-Me** + Me_3NO mimicking the conditions at 75% conversion (see Experimental Section for details). Spectrum was taken at rt after 5 minutes at rt. No peaks were observed up to 15 ppm or down to –30 ppm other than those shown.

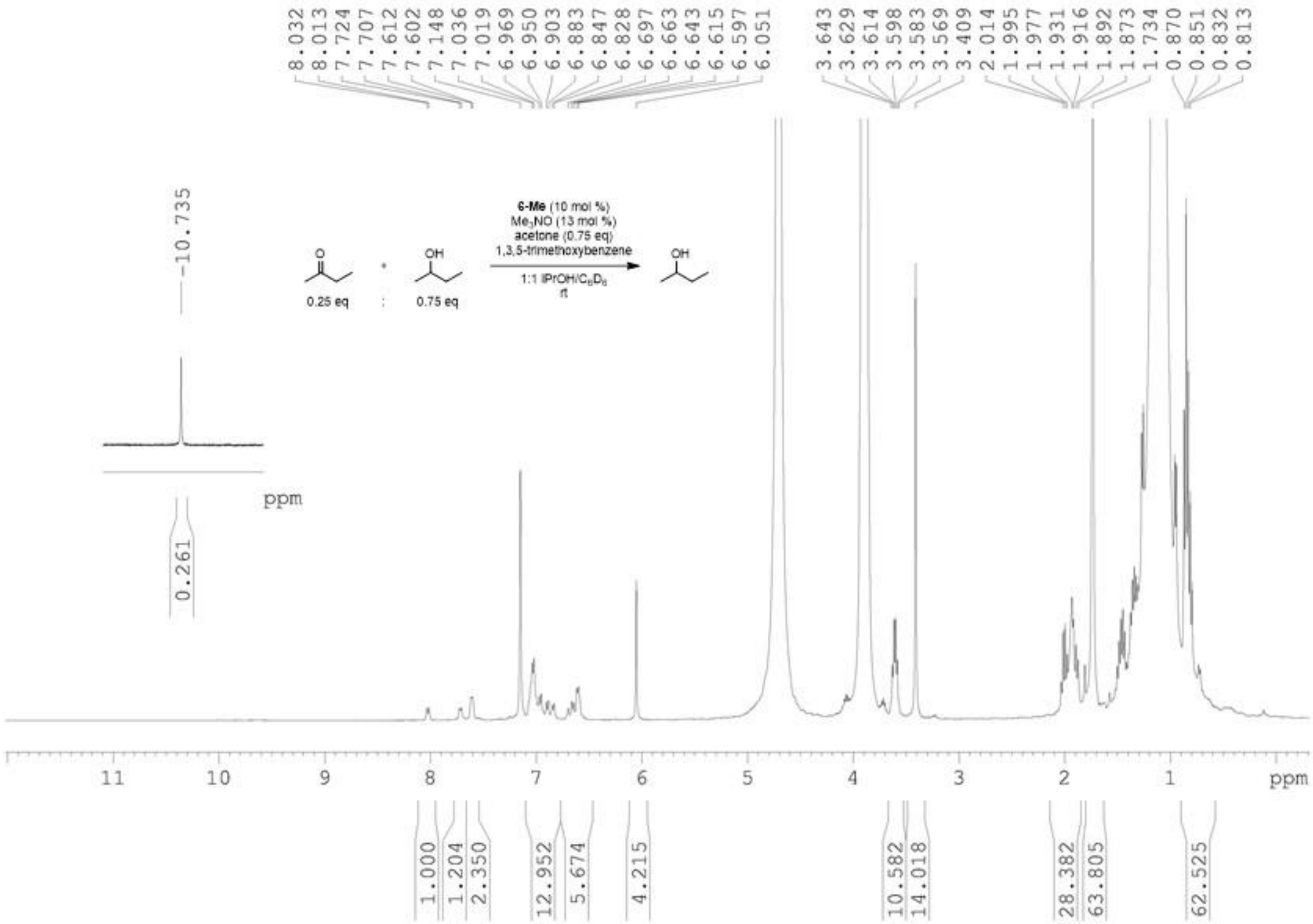

Figure S28. ^{1}H NMR spectrum (400 MHz, 1:1 benzene-d_6 and isopropanol) of transfer hydrogenation of 2-butanone with **6-Me** + Me_3NO mimicking the conditions at 75% conversion (see Experimental Section for details). Spectrum was taken at 65 °C after 15 minutes at 65 °C. No peaks were observed up to 15 ppm or down to –30 ppm other than those shown.

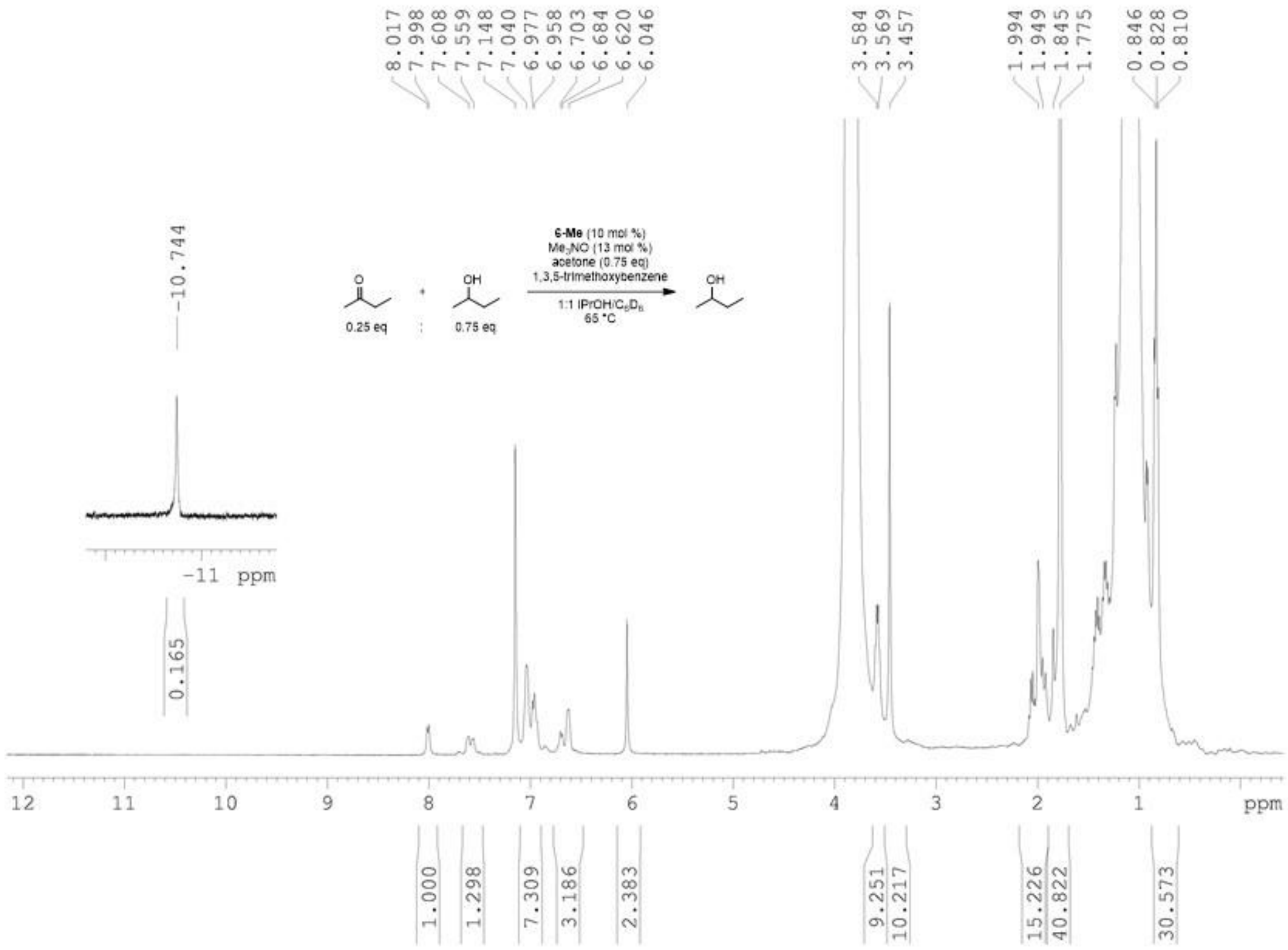

Supplement: Supplementary file 1 — om3c00284_si_001.pdf [file om3c00284_si_001.pdf]
